# Epidermal control of axonal attachment via β-spectrin and the GTPase-activating protein TBC-10 prevents axonal degeneration

**DOI:** 10.1038/s41467-019-13795-x

**Published:** 2020-01-09

**Authors:** Sean Coakley, Fiona K. Ritchie, Kate M. Galbraith, Massimo A. Hilliard

**Affiliations:** Clem Jones Centre for Ageing Dementia Research, Queensland Brain Institute, The University of Queensland, Brisbane, QLD 4072 Australia

**Keywords:** Cellular neuroscience, Molecular neuroscience

## Abstract

Neurons are subjected to strain due to body movement and their location within organs and tissues. However, how they withstand these forces over the lifetime of an organism is still poorly understood. Here, focusing on touch receptor neuron-epidermis interactions using *Caenorhabditis elegans* as a model system, we show that UNC-70/β-spectrin and TBC-10, a conserved GTPase-activating protein, function non-cell-autonomously within the epidermis to dynamically maintain attachment of the axon. We reveal that, in response to strain, UNC-70/β-spectrin and TBC-10 stabilize trans-epidermal hemidesmosome attachment structures which otherwise become lost, causing axonal breakage and degeneration. Furthermore, we show that TBC-10 regulates axonal attachment and maintenance by inactivating RAB-35, and reveal functional conservation of these molecules with their vertebrate orthologs. Finally, we demonstrate that β-spectrin functions in this context non-cell-autonomously. We propose a model in which mechanically resistant epidermal attachment structures are maintained by UNC-70/β-spectrin and TBC-10 during movement, preventing axonal detachment and degeneration.

## Abstract (web summary)

Neurons are subjected to strains due to body movements and their locations within organs and tissues. Here the authors show that UNC-70/β-spectrin and TBC-10 dynamically maintain attachment of the axon within the epidermis non-cell-autonomously by stabilizing hemidesmosome attachment, preventing axon degeneration.

## Introduction

Failure to maintain the integrity of the axon, the longest and most susceptible compartment of a neuron, results in compromised neuronal function, which is a characteristic of both traumatic injury and many neurodegenerative diseases^1^. Axons are subject to continuous strain, mostly due to body movement and their location within skin, muscles, organs, and joints. Excessive mechanical strain, or shear stress due to external or internal traumas, can trigger degeneration of the axon. Virtually every neuron, including those of the central nervous system, is susceptible to different types of strain insults^2,3^, and mechanical strain has been implicated in the progression of neurodegenerative disease^4,5^. Therefore, it is essential to understand the molecular mechanisms that protect against motion-induced injury in order to ensure that neurons and their axons maintain their correct structure and function throughout life.

**Fig. 1:**
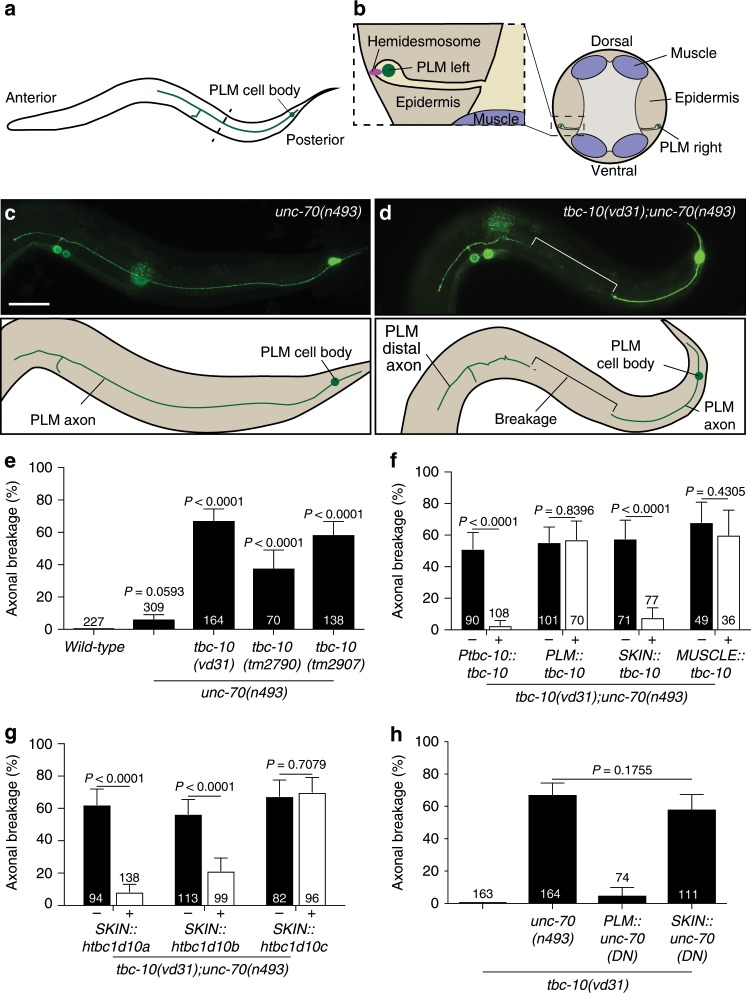
TBC-10 and UNC-70/β-spectrin function in the epidermis to protect against axonal breakage. **a** Scheme showing the location and morphology of the PLM touch receptor neuron. **b** A schematic transverse section through the animal (dotted line in **a**) illustrating the region in which the PLM axon is ensheathed in the epidermis and attached via hemidesmosomes. **c** An image and scheme showing a typical unbroken axon of the PLM neuron in *unc-70(n493)* mutants visualized with GFP (transgene *zdIs5(Pmec-4::GFP))*. **d** An image and scheme showing a typical broken axon of the PLM neuron in *tbc-10(vd31);unc-70(n493)* animals. **e** Mean penetrance of axonal breakage in wild-type, *unc-70(n493)* mutants, and multiple alleles of *tbc-10* in an *unc-70* background. **f** Mean penetrance of axonal breakage in *tbc-10;unc-70* mutants carrying transgenes expressing wild-type *tbc-10* under either an endogenous promoter, a touch receptor neuron-promoter, an epidermal-specific promoter, or a muscle-specific promoter, versus their respective non-transgenic siblings. **g** Mean penetrance of axonal breakage in *tbc-10(vd31);unc-70(n493)* mutants carrying transgenes expressing the wild-type human orthologs *tbc1d10a-c* under an epidermal-specific promoter versus non-transgenic siblings. **h** Mean penetrance of axonal breaks in *tbc-10* single and *tbc-10(vd31);unc-70(n493)* double mutants, versus *tbc-10(vd31)* mutants expressing single-copy insertions of the semi-dominant UNC-70(DN)::mKate2 driven by touch receptor neuron or epidermal-specific promoters. Scale bar in **c** = 50 μm. *P*-values in **e** and **h** are determined from a one-way ANOVA with a Tukey multiple-comparison of proportions using an Agresti–Coull interval. *P*-values in **f** and **g** are determined from a comparison of proportions using an Agresti–Coull interval. Error bars indicate a 95% confidence interval. *N*-values are indicated on bar graphs and represent the number of individual animals scored for each condition. Source data are provided as a Source Data file.

The cytoskeletal spectrin-network is thought to mediate strain resistance in axons by maintaining tension via the actin-spectrin cytoskeleton^6,7^. Human mutations in β-spectrin are associated with spinocerebellar ataxia, a neurodegenerative disorder that is characterized by uncoordinated gait, limb, and eye movement defects, slurred speech and swallowing difficulties^8^. In the nematode *C. elegans*, mutations in *unc-70/β-spectrin* cause motor and sensory axons to buckle and deform during body movements, leading to spontaneous axonal breakage^6,9,10^. In particular, specialized touch receptor neurons require a functional spectrin-network to maintain tension within the axon in order to transduce mechanical force, and limit buckling of the axon during normal body movement^6,7^. During normal development, the axons of the lateral touch receptor neurons ALM and PLM become ensheathed within the epidermis (Fig. 1a, b), and are mechanically coupled to this tissue via epithelial attachment structures along the axon, similar to invertebrate and vertebrate hemidesmosomes^11,12^. Proper attachment between the PLM neuron and surrounding epithelium is required for both the development and maintenance of touch receptor neurons^11,13^. Here, we identify a function for UNC-70/β-spectrin, the Rab-GTPase RAB-35, and its GTPase-activating protein (GAP) TBC-10, in preserving the axonal structure of *C. elegans* touch receptor neurons. We provide evidence that all three molecules function non-cell-autonomously within the surrounding epidermis to stablize hemidesmosomes against mechanical strain, and preserve the integrity of the ensheathed PLM axon by maintaining normal axon-epidermal attachment during movement. We show that the loss of hemidesmosomes in localized regions, and detachment of the axon from the epidermis in *tbc-10;unc-70* mutants, is followed by breakage and subsequent degeneration of the axon. Finally, we reveal a high level of functional conservation with the human orthologs of TBC-10 and RAB-35. Our work establishes a previously unknown, non-cell-autonomous and synergistic function of β-spectrin, RAB-35 and TBC-10 in dynamically regulating axonal maintenance by maintaining mechanically resistant hemidesmosomes and axon-epidermal attachment. Our results highlight a direct molecular link between neurons and their surrounding tissue that is critical for axonal maintenance, but dispensable for development.

## Results

### TBC-10 protects against spectrin-induced damage

*C. elegans unc-70/β-spectrin* is required to maintain neuronal integrity and to resist mechanical strain^6,7,9,10^. The spectrin-network functions both cell-autonomously within the PLM neuron, and non-cell-autonomously in the surrounding epidermis to limit buckling due to compressive forces^6^. Animals carrying a semi-dominant mutation in the αβ-tetramization domain of UNC-70/β-spectrin, *unc-70(n493)*, present with largely normal PLM neurons (Fig. 1c), but display a very low penetrance of axonal breakage not present in wild-type animals (Fig. 1e). To identify genes that protect against mechanically induced axonal damage, we performed an unbiased forward genetic screen using *unc-70* mutants with a low penetrance of axonal damage as a sensitized background (see methods for details). From this screen, we identified a mutant allele of the highly conserved gene *tbc-10*, an ortholog of human *tbc1d10a-c*, which encodes a Rab-GAP^14,15^. *tbc-10(vd31)* mutants exhibited a dramatic increase in axonal breakage of the PLM neurons in the *unc-70(n493)* mutant background (Fig. 1d, e). Two additional deletion alleles of *tbc-10* (*tm2790* and *tm2907*) both increased the rate of PLM axonal breakage more than fivefold compared to *unc-70* mutants (Fig. 1e). This enhancement of axonal breakage in the *tbc-10;unc-70* double mutants was rescued in transgenic animals carrying the wild-type *tbc-10* genomic locus with its upstream regulatory region (Fig. 1f). In a wild-type background, *tbc-10* mutant animals presented no spontaneous axonal breakage defects (Fig. 1h). Taken together these data demonstrate that TBC-10 functions to limit axonal damage in an *unc-70-*induced injury model.

### TBC-10 and UNC-70/β-spectrin function in the epidermis

To determine where *tbc-10* is expressed, we used a cytosolic GFP reporter under the control of a ~5 kb endogenous *tbc-10* regulatory region for visualization. We observed GFP expression within neurons, including PLM neurons, as well as in the epidermis, intestine, and excretory canal (Supplementary Fig. 1A–D). To identify where TBC-10 acts to mediate its protective effect, we generated transgenic strains carrying a wild-type copy of *tbc-10* driven by tissue-specific promoters. Tissue-specific expression of wild-type *tbc-10* in either PLM neurons or muscles, was not able to rescue the increase in axonal breakage of *tbc-10;unc-70* double mutants (Fig. 1f). In contrast, expression of wild-type *tbc-10* under an epidermal-specific promoter fully rescued the axonal breakage phenotype (Fig. 1f), revealing a key role for TBC-10 within this tissue. To test if the function of *tbc-10* in maintaining PLM axonal integrity is conserved across species, we expressed a complementary DNA (cDNA) encoding each of the three human orthologs, *tbc1d10a, tbc1d10b* and *tbc1d10c*, tissue-specifically in the epidermis of *tbc-10;unc-70* double-mutant animals (Fig. 1g). Remarkably, human *tbc1d10a* and *tbc1d10b* were able to rescue the axonal breakage phenotype of the double mutants (Fig. 1g), revealing a highly conserved function of these molecules throughout evolution. *tbc-10* shares more sequence homology with *tbc1d10a* (38%) and *tbc1d10b* (40%), whereas *tbc1d10c* (30%) is less similar. Moreover, within the critical TBC-domain *tbc1d10a* and *tbc1d10b* share 81% sequence identity with each other compared to 60% with *tbc1d10c*^16^. Taken together these results demonstrate that TBC-10 functions within the epidermis to protect the axons of PLM neurons from damage caused by disruption to UNC-70/β-spectrin, and shares functional conservation with its human orthologs TBC1D10A and TBC1D10B.

To determine whether the axonal protective effect of TBC-10 is specific for spectrin-induced injuries or more generally protects from damaging factors, we challenged *tbc-10* single-mutant animals with other insults that cause axonal injury. Firstly, we found that compared to wild-type animals, *tbc-10* mutants did not present with an increase in the rate of axonal degeneration in severed axon segments following laser-induced axotomy (Supplementary Fig. 2A). Furthermore, *tbc-10* mutants did not display increased axonal degeneration after alteration of microtubule stability in animals lacking the α-tubulin acetyltransferase MEC-17/αTAT1 (Supplementary Fig. 2B), or after treatment with colchicine or paclitaxel to destabilize and stabilize microtubules, respectively (Supplementary Fig. 2C)^17^. These results suggest that the axonal protective function of TBC-10 may be specific for spectrin-induced injury.

The cytoskeletal spectrin-network forms tetramers composed of α- and β-spectrin aligned head-to-head^18^. The *n493* allele of *unc-70/β-spectrin* contains an L2044P substitution in its 17^th^ spectrin repeat, which is proposed to affect αβ-tetramerization^6^ and functions in a semi-dominant manner. Given the requirement of UNC-70/β-spectrin dysfunction for axonal breakage to occur, we tested whether UNC-70/β-spectrin was also acting in the surrounding epidermis to protect the axon from damage. We used a modified *Mos1* transposon (*miniMos*)-mediated strategy^19^ to generate a transgenic strain carrying a single-copy of the semi-dominant *unc-70(n493)* allele tagged with mKate2 (*unc-70(dn)::mKate2*) and expressed selectively in either the epidermis or the touch receptor neurons, in the *tbc-10* mutant background (Fig. 1h). Tissue-specific expression of UNC-70(dn)::mKate2 in the epidermis of *tbc-10* mutant animals was sufficient to replicate the axonal breakage defect observed in the *tbc-10;unc-*70 double mutants, whereas selective expression of the same dominant allele in the touch receptor neurons did not cause axonal breakage (Fig. 1h). Thus, UNC-70/β-spectrin functions in the epidermis in synergy with TBC-10 to protect the PLM axon from damage.

To test if the UNC-70/β-spectrin-induced defect in *tbc-10* mutants was due to defects in tetramerization with α-spectrin, we over-expressed a dominant-negative version of SPC-1/α-spectrin (SPC-1(dn)) containing the tetramerization domain^6^ in either the epidermis, or touch receptor neurons. Expression of *spc-1(dn)* in either tissue replicated buckling defects previously described in spectrin mutants (Supplementary Fig. 3B)^6^, indicating it may have disrupted spectrin tetramerization as hypothesized, however it was not sufficient to cause axonal breakage in a *tbc-10* mutant background when expressed in either tissue (Supplementary Fig. 3A). Taken together these data suggest that together with TBC-10, UNC-70/β-spectrin functions in the epidermis to protect the ensheathed PLM neurons from mechanical damage, and this function may be independent of α-spectrin.

### TBC-10 and UNC-70/β-spectrin localize to the membrane

**Fig. 2:**
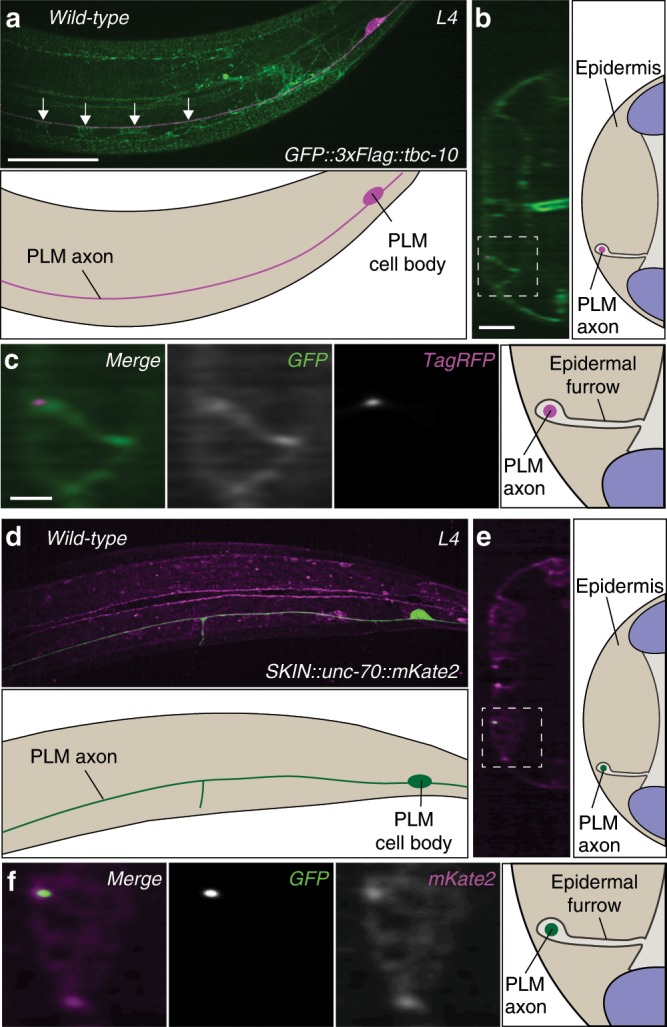
TBC-10 and UNC-70/β-spectrin localize to the epidermal membrane. **a** Deconvolved spinning disk confocal maximum projection and scheme of GFP::3xFlag::TBC-10 localization in a typical wild-type animal with the PLM neuron labeled with a cytosolic TagRFP (*Pmec-17::tagRFP*; magenta). Arrows indicate regions of the epidermal furrow visible in a lateral perspective. **b** An orthogonal perspective and scheme of GFP::3xFlag::TBC-10 localization in the projected *z*-stack shown in **a**. **c** A high-magnification image and scheme of the boxed area in **b**. These micrographs are representative of the GFP::3xFlag::TBC-10 localization seen in *N* = 12 animals at the L4 stage. **d** Image and scheme of UNC-70::mKate2 localization (magenta) expressed under an epidermal-specific promoter in a typical wild-type animal with the PLM neuron labeled with a cytosolic GFP (*Pmec-4::GFP)*. **e** An orthogonal perspective and scheme of UNC-70::mKate2 localization in the projected *z*-stack shown in **d**. **f** A high-magnification image and scheme of the boxed area in **e**. These micrographs are representative of the UNC-70::mKate2 localization seen in *N* = 6 animals at the L4 stage. Scale bars are 25 μm in **a**, 5 μm in **b**, and 2 μm in **c**.

To understand how TBC-10 protects the PLM axon from *unc-70-*induced damage, we engineered an N-terminal *gfp::3xFlag* tag at the endogenous *tbc-10* genomic locus using CRISPR-cas9 genome editing^20^. Using spinning disk confocal microscopy with deconvolution we visualized GFP::3xFlag::TBC-10 in large *z*-stacks, which were then rotated and viewed in an orthogonal plane to reveal the localization pattern of GFP::3xFlag::TBC-10 relative to the PLM neuron (labeled with a *Pmec-17::tagRFP* cell-specific marker; Fig. 2a–c). Importantly, the introduction of this tag did not cause axonal breakage in an *unc-70(n493)* background indicating that it is functional (Supplementary Table 1). In wild-type animals at the L4 stage, GFP::3xFlag::TBC-10 localized to the plasma membrane of the epidermis with a distinct and bright enrichment in a region directly apposed to the PLM axon, which corresponds to the epidermal furrow in which the PLM axon is ensheathed (Fig. 2a–c). An identical epidermal localization was observed in relation to the axon of the ALM neuron, which is also ensheathed within this tissue albeit in the dorsal, anterior region of the animal’s body (Supplementary Fig. 4).

Given the sensitivity of *tbc-10* mutants to UNC-70/β-spectrin dysfunction, we visualized the localization of UNC-70(wt)::mKate2 in the epidermis of wild-type animals. Using the above *miniMos*-mediated strategy, we generated a transgenic strain containing a single-copy insertion of a transgene expressing *unc-70(wt)::mKate2* under an epidermal-specific promoter and visualized its localization. This construct was able to partially rescue the breakage phenotype in *tbc-10;unc-70* animals, suggesting that it is functional (Supplementary Fig. 3C). In wild-type animals, UNC-70(wt)::mKate2 localized to the epidermal membrane at the lateral midline where two epithelial tissues, the hypodermal *hyp7* syncytium and the lateral seam cells, form adherens junctions (Fig. 2d). It also localized to a small number of puncta distributed throughout the epidermis between the apical and basal membranes (Fig. 2d–f), as well as a large punctate structure adjacent to the PLM axon and the apical surface of the epidermis when viewed in an orthogonal section (Fig. 2f), close to the site where hemidesmosomes form trans-epidermal attachments. Strikingly, overexpression of UNC-70(wt)::mKate2 in the *tbc-10* mutant background was sufficient to induce axonal breakage, although at a reduced rate compared to the overexpression of UNC-70(dn)::mKate2 (Fig. 1h) or the *unc-70(n493)* allele (Supplementary Fig. 3C). This phenotype was confirmed using a non-tagged version of wild-type UNC-70/β-spectrin expressed tissue-specifically in the epidermis (Supplementary Fig. 3D). A null allele of *unc-70/β-spectrin* in combination with *tbc-10* mutants also caused an increase in the rate of axonal breakage (Supplementary Fig. 3E). In these animals, the increased rate of axonal breakage was less than in those carrying other alleles of *unc-70*, an effect likely due to the reduced movement and very uncoordinated phenotype caused by the null allele. Indeed, consistent with this notion, RNAi-knockdown of *unc-70* by feeding in *tbc-10* mutants, which did not strongly affect movement, fully reproduced the axonal breakage defect (Supplementary Fig. 3F). This supports a model in which TBC-10 and UNC-70/β-spectrin are functioning at the epidermal membrane close to PLM to protect the ensheathed axon from damage. Furthermore, our data suggests that disruption to UNC-70/β-spectrin in any form is sufficient to affect its function in this context.

### TBC-10 regulates RAB-GTPase RAB-35 activity to protect axons

**Fig. 3:**
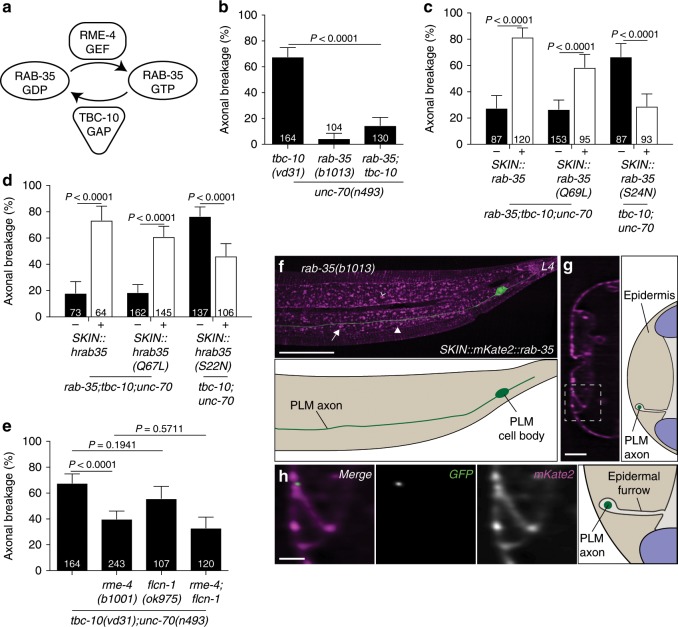
TBC-10 is a RAB-35-specific GAP. **a** Scheme of the activation cycle of RAB-35. **b** Mean penetrance of axonal breaks in *unc-70(n493)* mutants with and without mutations in *tbc-10(vd31)* and *rab-35(b1013)*. **c** Mean penetrance of axonal breakage in *rab-35(b1013);tbc-10(vd31);unc-70(n493)* or *tbc-10(vd31);unc-70(n493)* mutants carrying transgenes expressing either the wild-type *C. elegans rab-35*, a constitutively active *rab-35(Q69L)* mutant, or a constitutively inactive *rab-35(S24N)* mutant under the control of an epidermal-specific promoter, versus respective non-transgenic siblings. **d** Mean penetrance of axonal breakage in *rab-35(b1013);tbc-10(vd31);unc-70(n493)* or *tbc-10(vd31);unc-70(n493)* mutants carrying transgenes expressing either the wild-type human *rab35*, a constitutively active human *rab35(Q67L)* mutant, or a constitutively inactive human *rab35(S22N)* mutant under the control of an epidermal-specific promoter, versus their respective non-transgenic siblings. **e** Mean penetrance of axonal breaks in *tbc-10(vd31);unc-70(n493)* mutants, with and without single or double mutants for *rme-4* and *flcn-1*. *P*-values in **b** and **e** are determined from a one-way ANOVA with a Tukey multiple-comparison of proportions using an Agresti–Coull interval. *P*-values in **c** and **d** determined from a comparison of proportions using an Agresti–Coull interval. Error bars indicate a 95% confidence interval. Source data are provided as a Source Data file. **f** Deconvolved spinning disk confocal maximum projection and scheme of mKate2::RAB-35 localization (magenta) expressed under an epidermal-specific promoter in a typical *rab-35* mutant animal with the PLM neuron labeled with a cytosolic GFP (*Pmec-4::GFP*). Tubular, punctate, and spherical structures labeled with an arrow, closed arrowhead, and open arrowhead respectively. **g** An orthogonal perspective and scheme of mKate2::RAB-35 localization in the projected *z*-stack shown in **f**. **h** A high-magnification image and scheme of the boxed area in **g**. These micrographs are representative of the mKate2::RAB-35 localization seen in *N* = 5 animals at the L4 stage. Scale bars are 25 μm in **f**, 5 μm in **g**, and 2 μm in **h**. *N*-values are indicated on bar graphs and represent the number of individual animals scored for each condition.

RAB proteins are small GTPases that function as molecular switches to control a variety of intracellular trafficking pathways. Their activity is tightly controlled by the hydrolysis of GTP assisted by their specific GAPs and guanine exchange factors (GEFs)^21^ (Fig. 3a). TBC-10 is a RAB-specific GAP which, in other contexts, functions with specificity for RAB-35 (Fig. 3a)^22,23^. We predicted that in the absence of functional TBC-10 there would be an increase in the active pool of RAB-35-GTP. If this was the case, and TBC-10 was functioning as a RAB-35-specific Rab-GAP, a loss-of-function mutation in *rab-35* would protect against degeneration in *tbc-10;unc-70* double mutants. Indeed, we found that a *rab-35* mutant strongly suppressed the axonal breakage phenotype of the *tbc-10;unc-70* double mutants (Fig. 3b). These results reveal that TBC-10 functions as a RAB-35-specific Rab-GAP and inactivates RAB-35 to protect touch receptor neurons from damage, in synergy with UNC-70/β-spectrin.

Given that both TBC-10 and UNC-70/β-spectrin are specifically required in the epidermis, we expected that RAB-35 would also function in this tissue. To determine if this was the case, we tissue-specifically expressed a wild-type *rab-35* cDNA in the epidermis of *rab-35;tbc-10;unc-70* triple mutants. As predicted, this transgene fully restored the axonal breakage phenotype (Fig. 3c), revealing that RAB-35 also functions within the epidermis. To determine if this function of RAB-35 was conserved, we heterologously expressed a wild-type cDNA of human rab35 selectively in the epidermis. Remarkably, the human ortholog also fully restored the axonal breakage phenotype, suggesting that the function of RAB-35 is conserved across species (Fig. 3d).

To demonstrate that TBC-10 normally functions to increase the GTPase activity of RAB-35, we tested the effect of expressing predicted GTP- and GDP-locked versions of RAB-35 on axonal breakage. Tissue-specific overexpression of RAB-35(Q69L), predicted to lock RAB-35 in the GTP-bound conformation, in the epidermis of *rab-35;tbc-10;unc-70* triple mutants restored the axonal breakage phenotype (Fig. 3c). Furthermore, overexpression of a RAB-35(S24N) GDP-locked form tissue-specifically in the epidermis of *tbc-10;unc-70* mutant animals was able to rescue the axonal breaks (Fig. 3c). Remarkably, we oberved identical effects of GTP- and GDP-locked versions of human RAB35 when expressed heterologously in the epidermis (Fig. 3d). These data are consistent with TBC-10 functioning as a RAB-35-specific GAP in this context. In vivo, conversion of RAB-35-GDP to RAB-35-GTP is catalyzed by specialized GEFs. In *C. elegans* RME-4 and FLCN-1, orthologs of mammalian connecden and folliculin, respectively^14^, have been demonstrated to function as GEFs for RAB-35 (Fig. 3a)^22–24^. In the *tbc-10;unc-70* background, loss of function in *rme-4* partially rescued the axonal breakage phenotype, whereas *flcn-1* loss-of-function had no effect (Fig. 3e). Axonal breakage in *flcn-1;rme-4* double mutants in the *tbc-10;unc-70* background was indistinguishable from *rme-4* mutants, suggesting RME-4 functions as the major GEF for RAB-35 in this context (Fig. 3e). Given the partial rescue of *rme-4* loss-of-function, we cannot exclude the role in this context of another unknown GEF, in addition to RME-4. These data confirm TBC-10 and RME-4 function to regulate RAB-35 activity within the epidermis, which when overactive leads to axonal breakage in an *unc-70* mutant background.

We next asked where RAB-35 localized within the epidermis to modulate the axonal damage seen in *tbc-10;unc-70* double mutants. To investigate this we expressed and visualized RAB-35::mKate2 selectively in the epidermis of *rab-35(b1013)* mutant animals (Fig. 3f–h). RAB-35::mKate2 was enriched in punctate, tubular and spherical structures close to the apical membrane of the epidermis and, similar to TBC-10, at the epidermal furrow (Fig. 3f–h). Therefore, RAB-35 is normally localized close to the PLM axon in the epidermal membrane, is enriched in the furrow surrounding the PLM axon, and TBC-10 loss-of-function sensitizes the axon to spontaneous breakage by increasing the active pool of RAB-35-GTP.

### UNC-70/β-spectrin and TBC-10 regulate hemidesmosomes

**Fig. 4:**
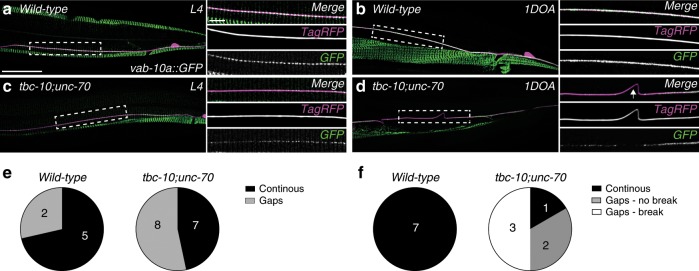
VAB-10a/spectraplakin localization is disrupted in *tbc-10;unc-70* mutants. **a**, **b** Deconvolved spinning disk confocal maximum projection of typical endogenous VAB-10a::GFP localization at the L4 (**a**) and 1-day-old adult (1DOA) (**b**) stage in wild-type animals from a lateral perspective, with the PLM neuron labeled with a cytosolic TagRFP (*Pmec-17::tagRFP*, magenta). The side panels in **a**–**d** show the boxed area indicated in the left panel as a merged image and individual channels. **c**, **d** Deconvolved spinning disk confocal maximum projection of typical endogenous VAB-10a::GFP localization at the L4 (**c**) and 1DOA (**d**) stage in *tbc-10(vd31);unc-70(n493)* animals from a lateral perspective, with the PLM neuron labeled with a cytosolic TagRFP (*Pmec-17::tagRFP*, magenta). Arrow in **d** indicates a buckle in the PLM axon. Scale bars in **a** are 25 μm and 5 μm in the left and side panels, respectively. **e** Normalized intensities of VAB-10a::GFP along a 50 μm of the PLM axon at the L4 stage were scored as containing gaps (defined as >5 puncta with intensity values < 0.2 a.u.) or continuous attachment (defined as < 5 puncta with intensity values < 0.2 a.u.). **f** VAB-10a::GFP localization at the 1DOA stage was scored as continuous (no gaps in localization greater than 10 μm) or containing gaps (a region of no localization greater than 10 μm) with and without axonal breakage. *N*-values are indicated on charts and represent the number of individual animals scored for each condition.

The pattern of TBC-10, UNC-70 and RAB-35 localization in regions of the epidermis overlying the muscle was reminiscent of fibrous structures, called hemidesmosomes, which mechanically couple the muscle to the cuticle via the epidermis (Fig. 2a, d and Fig. 3f)^25^. Similar structures also transmit mechanical deformation of the cuticle to the touch receptor neurons^25–29^. This led us to hypothesize that the attachment between the touch receptor neuron PLM and its surrounding epidermis via hemidesmosomes may be disrupted in *tbc-10;unc-70* mutants and drive the axonal breakage phenotype. To test this possibility, we visualized the localization of VAB-10a/spectraplakin, a core component of *C. elegans* hemidesmosomes and the sole ortholog of vertebrate plectin and BPAG1e^29^. In wild-type animals at the L4 stage, a functional CRISPR-Cas9 edited VAB-10a::GFP localized to epidermis-muscle and axon-epidermis attachment sites in a circumferentially striped and periodic pattern (Fig. 4a and Supplementary Fig. 5A–D) as previously reported^29,30^. Twenty-four hours later, in 1-day-old adult (1DOA) animals, the periodicity of VAB-10a::GFP localization was lost and VAB-10a::GFP was continuously distributed along the length of the PLM axon in wild-type animals (Fig. 4b and Supplementary Fig. 5A–D). This change in localization pattern may reflect a remodeling event within hemidesmosomes at this stage. As predicted, *tbc-10;unc-70* mutants presented with defects in the attachment of the PLM axon to the surrounding epidermis. This was evident in the L4 stage before the appearance of axonal breakage, where there was an increase in the proportion of animals missing VAB-10a::GFP-positive puncta along the length of the axon in localized regions compared to wild-type animals (Fig. 4c, e). The defect worsened in 1DOA animals where the localization of VAB-10a::GFP became discontinuous and large gaps appeared in regions of intact and broken PLM axons (Fig. 4d, f). In the example shown in Fig. 4d, we observed a region of almost complete loss of VAB-10a::GFP combined with buckles in the PLM axon. Importantly, we did not observe buckles or breaks in regions with continous attachment, nor did we observe any changes to the periodicity or spacing of VAB-10a::GFP-positive puncta in regions where attachment was present (Supplementary Fig. 5B, C). Single mutants for *tbc-10* and *unc-70* had normal attachment (Supplementary Fig. 6A–F), although the levels of VAB-10a::GFP were reduced in *unc-70* single mutants compared to wild-type animals in 1DOA (Supplementary Fig. 5B). These data demonstrate that hemidesmosome attachment structures are disrupted in *tbc-10;unc-70* axons prior to axon breakage and partially lost in adult animals.

**Fig. 5:**
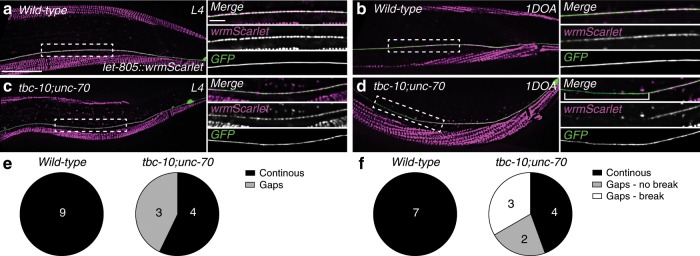
LET-805/myotactin localization is disrupted in *tbc-10;unc-70* mutants. **a**, **b** Deconvolved spinning disk confocal maximum projection of typical endogenous LET-805::wrmScarlet localization (magenta) at the L4 (**a**) and 1-day-old adult (1DOA) (**b**) stage in wild-type animals from a lateral perspective, with the PLM neuron labeled with a GFP (*Pmec-4::GFP*). The side panels in **a**–**d** show the boxed area indicated in the left panel as a merged image and individual channels. **c**, **d** Deconvolved spinning disk confocal maximum projection of typical endogenous LET-805::wrmScarlet localization (magenta) at the L4 (**c**) and 1DOA (**d**) stage in *tbc-10;unc-70* animals from a lateral perspective, with the PLM neuron labeled with a cytosolic GFP (*Pmec-4::GFP*). Open bracket in side panel **d** indicates the region where LET-805::wrmScarlet localization is absent. Scale bars in **a** are 25 μm and 5 μm in the left and side panels, respectively. **e** Normalized intensities of LET-805::wrmScarlet along a 50 μm of the PLM axon at the L4 stage were scored as containing gaps (defined as >5 puncta with intensity values < 0.2 a.u.) or continuous attachment (defined as < 5 puncta with intensity values < 0.2 a.u.). **f** LET-805::wrmScarlet localization at the 1DOA stage was scored as continuous (no gaps in localization greater than 10 μm) or containing gaps (a region of no localization greater than 10 μm) with and without axonal breakage. *N*-values are indicated on charts and represent the number of individual animals scored for each condition.

In addition to the VAB-10a::GFP localization, we investigated the localization of LET-805/myotactin, a key adhesion molecule containing multiple fibronectin repeats predicted to link VAB-10a to integrins on the muscle and the PLM axon on the basal side of the epidermis^27,31^. Consistent with our VAB-10a findings, a CRISPR-Cas9 edited *let-805::wrmScarlet* localized to periodic puncta along the PLM axon in L4 stage wild-type animals (Fig. 5a and Supplementary Fig. 5E–H). The tagged LET-805::wrmScarlet was deemed functional as animals were viable. In 1DOA wild-type animals the periodic localization was lost, with a more uniform and continuous distribution observed along the axon (Fig. 5b and Supplementary Fig. 5E–H). As with VAB-10a, LET-805::wrmScarlet localization revealed a loss of attachment of the PLM axon to the surrounding epidermis in *tbc-10;unc-70* animals. At the L4 stage there was an increase in the proportion of animals missing LET-805::wrmScarlet-positive puncta along the length of the axon in localized regions compared to wild-type animals (Fig. 5c, e). In 1DOA *tbc-10;unc-70* animals we observed large gaps in the localization of LET-805::wrmScarlet in intact and broken PLM axons (Fig. 5d, f). In *tbc-10* and *unc-70* single mutants, LET-805::wrmScarlet was localized along the PLM axon in an identical pattern to *wild-type* animals (Supplementary Fig. 6G–L). Interestingly, each single mutant had opposing effects on the membrane levels of LET-805::wrmScarlet in each stage. At the L4 stage *unc-70* animals had a reduced intensity of LET-805::wrmScarlet (Supplementary Fig. 5F), while in 1DOAs *tbc-10* mutants displayed an increase in the level of LET-805::wrmScarlet at the membrane compared to wild-type animals (Supplementary Fig. 5F).

Combined with the strong genetic data presented, these results indicate that *tbc-10* and *unc-70* mediate parallel pathways in maintaining attachment of the PLM axon to the surrounding epidermal tissue, which is critical for axonal integrity. Importantly, the disruption observed in the localization of both VAB-10a::GFP and LET-805::wrmScarlet in *tbc-10;unc-70* double-mutant animals at the L4 stage preceded, and was predictive of, future breakage events (Supplementary Fig. 6M–P). Therefore our results support a model in which the PLM axon breaks as a consequence of the partial loss of hemidesmosome mediated attachment between the axon and its surrounding epidermis.

### Strain of movement induces loss of hemidesmosomes in mutants

**Fig. 6:**
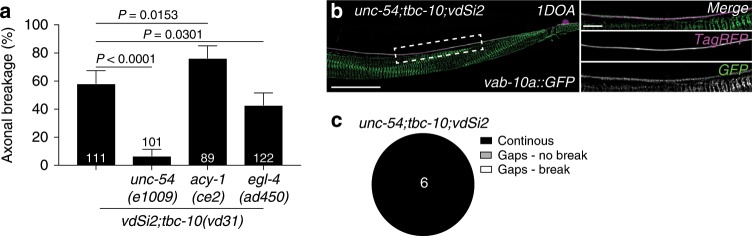
Attachment defects and axonal breakage are movement dependent. **a** Mean penetrance of axonal breaks in *tbc-10(vd31);vdSi2(Pdpy-7::mKate2::unc-70(n493))* animals with and without mutations in *unc-54, acy-1, and egl-4*. **b** Deconvolved spinning disk confocal maximum projection of typical endogenous VAB-10a::GFP localization in 1-day-old adult (1DOA) *unc-54;tbc-10;vdSi2* animals from a lateral perspective, with the PLM neuron labeled with a cytosolic TagRFP (*Pmec-17::tagRFP*; magenta). The side panels in **b** show the boxed area indicated in the left panel as a merged image and individual channels. Scale bars in **b** are 25 μm and 5 μm in the left and side panels, respectively. **c** VAB-10a::GFP localization at the 1DOA stage was scored as continuous (no gaps in localization greater than 10 μm) or containing gaps (a region of no localization greater than 10 μm) with and without axonal breakage. *P*-values in **a** are determined from a one-way ANOVA with a Tukey multiple-comparison of proportions using an Agresti–Coull interval. Error bars indicate a 95% confidence interval. *N*-values are indicated on bar and pie charts and represent the number of individual animals scored for each condition. Source data are provided as a Source Data file.

**Fig. 7:**
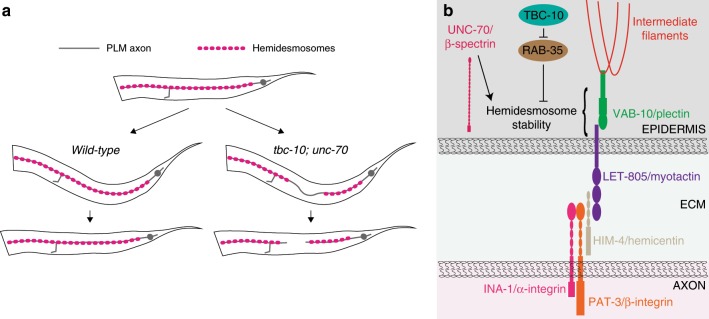
Proposed model for attachment defects leading to axonal degeneration. **a** During normal body movement hemidesmosomes are mechanically resistant and attachment is maintained along the length of the axon in wild-type animals. In *tbc-10;unc-70* mutants, these attachments are no longer mechanically resistant and the strain of movement leads to the intermittent loss of hemidesmosomes. Successive rounds of movement leads to increased local strain in the now detached axon, causing axonal breakage and degeneration. **b** Schematic representation of known and proposed components of hemidesmosomes linking the axons of touch receptor neurons to the epidermis. We propose that UNC-70/β-spectrin, the Rab-GTPase-activating protein TBC-10 and its RAB-35 target, maintain axonal integrity by functioning non-cell autonomously in the epidermis to control the stability of hemidesmosomes in response to mechanical strain via parallel pathways.

*C. elegans* hemidesmosomes have been suggested to function as mechanosensors during epithelial morphogenesis and respond to tension by activating signaling pathways^32^. To confirm that the axonal breaks were induced by the mechanical strain of movement, we took advantage of *unc-54(e1009)* mutant animals, which carry a mutation in a muscle myosin class II heavy chain and as a consequence are severely paralyzed. In the absence of movement, the axons of *unc-54;tbc-10;vdSi2(Pdpy-7::unc-70(dn)::mKate2)* animals did not break, suggesting that the axonal breakage phenotype is a consequence of movement (Fig. 6a). Consistent with this model, a gain-of-function mutation in the adenylate cyclase *acy-1* that induces a hyperactive phenotype^33^ was able to increase the rate of axonal breakages in *tbc-10;vdSi2(Pdpy-7::unc-70(dn)::mKate2)* animals (Fig. 6a). Conversely, a gain-of-function mutation in the cGMP-dependent protein kinase gene *egl-4*, which causes increased periods of quiescence^34^, reduced the rate of axonal breakage in *tbc-10;vdSi2(Pdpy-7::unc-70(dn)::mKate2)* animals (Fig. 6a). In principle, defects in hemidesmosomes could arise independently of movement as a consequence of UNC-70/β-spectrin and TBC-10 dysfunction, and movement provides only the mechanical force required to induce damage in detached axons. Alternatively, UNC-70/β-spectrin and TBC-10 may function to ensure hemidesmosomes are mechanically resistant. In this case loss of hemidesmomes would only occur following movement. To distinguish between these possibilities we visualized VAB-10a::GFP localization in *unc-54;tbc-10;vdSi2(Pdpy-7::unc-70(dn)::mKate2)* paralyzed animals. Intriguingly, VAB-10a::GFP attachment sites remained intact and continuous in paralyzed animals at the 1DOA stage (Fig. 6b, c). These data are consistent with UNC-70/β-spectrin and TBC-10 functioning within the epidermis to maintain mechanically resistant hemidesmosomes and axon-epidermis attachment during movement (Fig. 7).

## Discussion

To maintain a functional nervous system, circuits must be able to withstand varying degrees of mechanical strain during their lifetime. Using a forward genetic approach we have identified a key protection mechanism for a *C. elegans* sensory axon that allows their attachment to be maintained during mechanical strain and protects from strain-induced degeneration. We have several lines of evidence to support this model. Firstly, we show that *tbc-10;unc-70* mutants undergo spontaneous axonal degeneration in response to normal body movement. Secondly, we demonstrate that UNC-70/β-spectrin and the Rab-GTPase-activating protein TBC-10 function non-cell autonomously and synergistically in the epidermis to maintain hemidesmosomes at sites of axon-epidermal attachment. Thirdly, loss of hemidesmosomes in *tbc-10;unc-70* mutants is induced by movement—indicating that their normal function is to maintain these fibrous organelles under conditions of increased strain.

The cytoskeletal spectrin-network is thought to mediate strain resistance intrinsically within axons by maintaining tension via the actin-spectrin cytoskeleton^6,7^. In the nematode *C. elegans*, disruption of the cytoskeletal spectrin-network causes axons to buckle and deform during body movement, leading to spontaneous axonal breakage^6,9,10^. Until recently, most data supported a model in which the spectrin-network functioned largely cell-autonomously within neurons to protect them from damage. However, it is becoming increasingly clear that β-spectrin has a much larger role in neuronal support cells than was previously appreciated. Here, we uncover a role for β-spectrin in the maintenance of axonal integrity, whereby it functions non-cell-autonomously in the epidermis surrounding a mechanosensory axon to regulate adhesion. We have some evidence to suggest this role is potentially independent of its teteramerization partner, α-spectrin, as the predicted disruption to the spectrin-network with a dominant-negative α-spectrin was not sufficient to cause axonal damage. There are multiple examples of β-spectrin interacting with integral membrane proteins and cell adhesion molecules^35^. β-spectrin directly interacts with the neural cell adhesion molecule NCAM^36,37^, as well as with CHL1, a close homolog of the immunoglobulin superfamily cell adhesion molecule L1^38^, and is required independently of α-spectrin for the polarized localization of the Na^+^/K^+^ ATPase in the epithelia of the *Drosophila* midgut^39^. On its own, disruption to *unc-70/β-spectrin* causes only a mild axonal breakage phenotype; however, in combination with mutations in the Rab-GAP *tbc-10*, and subsequent increases in RAB-35 activity, it causes loss of hemidesmosomes, detachment of the axon from the surrounding epidermis, and axonal degeneration. The PLM touch receptor neurons of *C. elegans* are unmyelinated and lack specialized glia. Instead, they have an intimate and complex relationship with the overlying epidermal tissue, which is essential for the correct development of the PLM neuron^11^ and, as we demonstrate here, the maintenance of its integrity. In this respect, the relationship resembles that of specialized myelinating glia, oligodendrocytes and Schwann cells, which wrap their membrane around axons to provide insulation and trophic support, sculpt structure, and cluster ion channels^40^. In *C. elegans*, the spectrin-network has been demonstrated to partially function non-cell-autonomously within the epidermis to limit buckling of touch receptor neuron axons during normal body movement, where it was postulated to affect membrane elasticity^6,7^. Moreover, in vertebrate myelinating glia the spectrin-based cytoskeleton has been implicated in the formation and maintenance of adhesion complexes at the paranodal regions flanking nodes of Ranvier^41,42^, and disruption of axon-glia attachments causes axonal degeneration in Purkinje neurons^43^. In this context, together with the data we present here, this suggests mechanisms governing adhesion play highly conserved roles in axonal protection.

Our results illuminate a complex and intricate system regulating tissue attachment and neuronal maintenance in response to movement-induced mechanical strain. In mutants where attachment pathways are completely disrupted, the PLM axon fails to become ensheathed within the epidermis and instead resides between the muscle and epidermis where it originally develops^11^. We have previously shown that preventing attachment and ensheathing of the PLM axon within the epidermis is not sufficient to cause axonal degeneration^44^. A potential explanation for these seemingly contradictory phenotypes is that in the case of uniform attachment (or uniform detachment) of the axon and epidermis, the axon is able to withstand compression from body movement due to intrinsic protection mechanisms. However, we propose that partial detachment may be detrimental because of an imbalance in tension and force experienced by the axon. Here, we demonstrate that following normal development, hemidesmosomes are susceptible to subtle changes, which render them, and consequently the ensheathed axon, vulnerable to mechanical stress and degeneration. The axonal process of PLM is coupled to the surrounding epithelia via mechanically resilient adhesion complexes, which act like shock absorbers to both transduce force from the cuticle to the axon for touch sensation^26^, as well as minimizing the damaging compressive forces experienced by the axon during body movement. Hemidesmosomes have been postulated to facilitate tissue sliding while maintaining normal attachment by dynamic assembly and disassembly during tissue movement^45^, and are highly susceptible to subtle perturbations in the levels of their components and the dynamics of their assembly and disassembly^46^. Hemidesmosomes have also been shown to act as mechanosensors during epithelial morphogenesis, relaying muscle tension to intiate intracellular signaling processes^32^ and are remodeled in response to stretch^47^. Importantly, loss of hemidesmomes and subsequent axonal breakage in *tbc-10;unc-70* mutants was prevented by paralysis. We propose that the mechanical strain of body movement is transmitted via hemidesmosomes into the epidermis, where TBC-10 and UNC-70/β-spectrin function redundantly to maintain mechanically resistant hemidesmosomes, potentially by participating in remodeling or stabilization of these structures to prevent their disassembly (Fig. 7b). Indeed, TBC-10 and RAB-35 have known roles in membrane recycling events^14,24^ and β-spectrin has been demonstrated to both stabilize membrane proteins in epithelia^39^ and adhesion complexes mediating axon-glia attachment^41–43^. Consistent with this notion, our results indicate that UNC-70 and TBC-10 can regulate the levels and balance of the hemidesmosome components on the membrane, with mutations in *unc-70* causing a decrease of both VAB-10 and LET-805, whereas mutations in *tbc-10* cause an increased level of LET-805. Our results paint a picture of axonal adhesion as a finely balanced and tightly regulated protective mechanism that contributes to the mechanical resilience of axonal compartments.

## Methods

### Strains

All nematodes were cultured on nematode growth medium (NGM) plates seeded with *Escherichia coli* OP50 according to standard methods^48^. Any strain containing the *unc-70(n493)* or *unc-70(s1502)* alleles were cultured in the same conditions with *Escherichia coli* HB101. Unless otherwise noted, experiments were performed on 1-day-old adults raised at 22 °C, selected 24 h prior at the L4 stage. The wild-type N2 Bristol strain was used with the alleles and transgenes listed in Supplementary Tables 1 and 2. The morphology marker *uIs115(Pmec-17::tagRFP)]* was a gift from Martin Chalfie, *unc-70(s1502)* and *oxIs95(Ppdi-2::unc-70)* were gifts from Erik Jorgensen, and *pgIs10[Pmec-17::spc-1(1-170)::mCherry]* was a gift from Miriam Goodman.

### Isolation of genetic mapping of mutant

Animals of genotype *unc-70(s1502);oxIs95(Ppdi-2::unc-70);zdIs5* were mutagenized using 50 mM ethyl methanesulfonate (EMS; Sigma) for 4 h. The *vd31* mutation was isolated from a non-clonal F2 progeny screen. The *vd31* allele was crossed into an *unc-70(n493);zdIs5* background and backcrossed x3 with N2 Bristol worms before whole-genome sequencing was conducted (Queensland Brain Institute, Center for Brain Genomics) on QH5436(*vd31;unc-70(n493);zdIs5*), QH3631(*unc-70(s1502);oxIs95;zdIs5*), QH5343(*unc-70(n493);zdIs5*) and the N2 Bristol strain at a mean depth of ≥58. Mapping and calling of variants was performed using CloudMap^49,50^ by comparing the frequency of likely EMS mutations across the genome^51^ after background subtraction.

### Molecular biology, CRIPSR-Cas9 gene editing, and single-copy insertions

Standard molecular biology techniques were used^52^. All plasmids were generated using either classic restriction enzyme digestion followed by ligation or Gibson assembly^53,54^ and details of their construction are listed in Supplementary Table 3.

Gene-edited *tbc-10* knock-in strains were generated using CRISPR-Cas9 gene editing with a self-excising cassette^20^. Approximately 650 bp homology arms were inserted into the GFP^SEC^3xFlag vector pDD282 (a gift from Bob Goldstein; Addgene plasmid #66823) using Gibson assembly (amplified from N2 genomic DNA). A Cas9 target site was chosen using the MIT CRISPR design tool (http://crispr.mit.edu) and inserted into a Cas9–sgRNA vector pDD162 (a gift from Bob Goldstein; Addgene plasmid #47549) using QuikChange Site-directed Mutagenesis (Agilent Technologies, USA). To generate lines, 10 ng/µL of the homologous repair template and 50 ng/µL of the Cas9–sgRNA construct containing targeting sequence, as well as 10 ng/μL pGH8, 5 ng/μL pCFJ104, and 2.5 ng/μL pCFJ90 (gifts from Erik Jorgensen; Addgene #19359, #19328 and #19327) were injected into the gonads of young adult animals. Injected animals were transferred to new OP50 plates (three per plate) and allowed to generate progeny for 3 days at 25 °C. Then, hygromycin was added to a final concentration of 250 µg/mL and placed at 25 °C for another 4 days. Animals that survived hygromycin selection, were rollers and lacked fluorescent injection markers were singled onto fresh OP50 plates to establish lines. A knock-in *let-805(syb380)* allele with a C-terminal wrmScarlet tag was obtained from Suny Biotech (China).

miniMos NeoR vectors were constructed using Gibson assembly of two PCR fragments to insert the construct of interest into the miniMos NeoR (pCFJ910 was a gift from Erik Jorgensen; Addgene plasmid #44481). Single-copy insertions^19^ were generated by injecting young adult animals with miniMos-based vectors at 10 ng/μL together with pGH8 10 ng/μL, pCFJ90 2.5 ng/μL, pCFJ104 10 ng/μL, pCFJ601 50 ng/μL, and pMA122 10 ng/μL (gifts from Erik Jorgensen; Addgene #34874 and #34873). Injected animals were transferred to new OP50 plates (three per plate) and allowed to generate progeny at 25 °C. One day later, 500 μL of 25 mg/mL G418 was added to each plate for NeoR selection (Thermo Fisher Scientific, USA). Plates were left to starve at 25 °C, and once starved were heatshocked at 34 °C in an air incubator to kill animals carrying extra-chromosomal arrays. Four hours after heat shock, plates containing animals that were alive, lacked co-injection markers and moved well were chunked onto a fresh OP50 plate and grown at 22 °C. Two days later, single, healthy animals containing successful insertions were selected and placed into individual OP50 plates.

### Phenotypic analysis

Axonal breakage was scored in 1-day-old adult animals, selected 24 h prior at the L4 stage in animals containing the *zdIs5(Pmec-4::GFP), uIs115(Pmec-17::tagRFP)* or *zdIs4(Pmec-4::GFP)* transgenes. The mean observed proportion of axonal breakage in each strain and 95% confidence interval was calculated using an Agresti–Coull estimation of binomial distribution.

### Laser axotomy

Laser axotomies were performed using a MicroPoint Laser System Basic Unit attached to a Zeiss Axio Imager A1 (Objective EC Plan-Neofluar 100x/1.30 Oil M27)^55^. This laser delivers 120 mJ of 337 nm energy with a 2 to 6 ns pulse length. Axotomies were completed with 10 to 20 pulses in both wild-type and *tbc-10(vd31)* mutant animals. The PLM axon of animals at the L4 stage was cut at a point ~50 μm anterior to the PLM cell body, and the resulting distal fragment was assessed for degeneration 24 h post-axotomy. Neurons that underwent post-axotomy axonal fusion were excluded from the experiment. The severity of degeneration and clearance was analyzed for each neuron using an arbitrary scale: 1 = fully cleared axon, 2 = multiple breaks, 3 = one break, 4 = beading and thinning, and 5 = intact axon^56^.

### Microscopy

Animals were inspected by mounting on 4% agar pads after anesthesia with 0.05% tetramisole hydrochloride. Fluorescence microscopy was performed using an upright Zeiss AxioImager A1 microscope equipped with a Photometrics Cool Snap HQ2 camera. Images acquired in Metamorph software were further processed in ImageJ v1.51r. Confocal imaging was performed at the Queensland Brain Institute’s Advanced Microscopy Facility using a spinning disk confocal microscope (Marianas, Intelligent Imaging Innovations, USA) equipped with a confocal scanner unit (CSU-W1, Yokogawa Electric Co., Japan) built around an Axio Observer body (Z1, Carl Zeiss AG, Germany) and fitted with an sCMOS camera (ORCA-Flash4.0 V2, Hamamatsu Photonics, Japan) and SlideBook v6.0 software (3i, USA) using a 100 × /1.46 NA oil-immersion objective with sampling intervals *x*,*y* = 63 nm and *z* = 130 nm. Images were deconvolved with Huygens Professional v18.04 run on a GPU-accelerated computer (3x NVIDIA® Tesla® V100) using the CMLE algorithm, with signal to noise ratio of 20, background of 100, and 40 iterations. Images exported as 32-bit TIFs and further processed in ImageJ v1.51r to obtain YZ-plane orthogonal views. Microinjections were performed using standard methods^57^, with an inverted Zeiss AxioObserver microscope equipped with differential interference contrast, a Narishige needle holder, and an Eppendorf FemtoJet pump.

### Image analysis

Quantification of puncta periodicity and intensity was performed using ImageJ v1.51r and Matlab (Mathworks, USA). Deconvolved confocal *z*-stacks acquired of the PLM neuron, including the neuronal cell body and the first 80–100 µm of the axon were converted into maximum image projections of 21 slices centered around the axon. The axon was traced using the ImageJ plugin NeuronJ and an ROI of this trace created for each image. A line scan using this trace as a mask was then obtained and the intensity values for each point extracted. Using Matlab, 793 intensity values for each image were extracted corresponding to a 50 µm region of each axon. All intensities were normalized to the mean intensity value observed in wild-type controls at the L4 stage. Autocorrelation curves were generated from these values to measure periodicity. The autocorrelation amplitude was defined as the difference between the first peak and the mean of the two valleys of the autocorrelation curve^58^. Mean puncta spacing was defined as the mean distance between intensity peaks across the region analyzed. Normalized intensities of each genotype at the L4 stage were scored as having gaps in attachment (defined as >5 puncta with intensity values < 0.2 a.u.) or continuous attachment (defined as < 5 puncta < 0.2 a.u.). Deconvolved maximum image projections of each genotype at the 1DOA stage were scored for the continuity of VAB-10a::GFP/LET-805::wrmScarlet along the PLM axon. Localization was scored as continuous (no gaps in localization > 10 μm) or containing gaps (a region of no localization for > 10 μm) with and without axonal breakage. All animals had a large gap in localizations near the anus, which was excluded from the analysis, only the anterior neurite was scored in all examples.

### Drug treatments

Animals were grown on NGM agar plates containing 0.1 mM colchicine (Sigma-Aldrich) or 1 µM paclitaxel (Sigma-Aldrich) in dimethyl sulfoxide (DMSO)^17,59,60^. For control plates, animals were grown with 1% DMSO. Parental (P0) animals were grown from the L4 stage on drug plates, and their F1 progeny were scored as 3-day-old adult animals selected at the L4 stage and scored 72 h later.

### RNAi

RNAi was induced by feeding^61^. Fifty milligram per milliliter Amplicillin, 12.5 mg/mL Tetracycline, and 1 mM isopropyl β-d-1-thiogalactopyranoside were added to standard NGM agar, poured into 35 mm petri dishes and allowed to dry overnight at room temperature. *E. coli* HT115 carrying either *L4440* control empty vector (*L4440* was a gift from Andrew Fire; Addgene plasmid #1654) or *unc-70* (clone K11C4.3, Ahringer library^62^) were grown in 2 mL of LB containing 50 mg/mL Amplicillin and 12.5 mg/mL Tetracycline at 37 °C with shaking for 7 h. Several drops of these cultures were then seeded onto the above modified NGM agar and allowed to dry and induce for 2 days. Eight adult animals of each genotype were added to each plate and allowed to lay eggs for 7 h before being removed. The progeny were then scored for axonal breaks 3 days later as young adults.

### Reporting summary

Further information on research design is available in the Nature Research Reporting Summary linked to this article.

## Supplementary information

Supplementary Information

Reporting Summary

## Data Availability

The datasets generated and analyzed for Figs. 1, 3, 6, and Supplementary Figs. 2 and 3 are available in the Source Data file. Datasets generated and analyzed for Figs. 4, 5, and 6 and Supplementary Figs. 5 and 6 and all other data are available from the corresponding author on reasonable request.

## Source data

Source Data File
