# Supplementary material for: Epidermal control of axonal attachment via β-spectrin and the GTPase-activating protein TBC-10 prevents axonal degeneration
Source: Nat Commun. 2020 Jan 9;11:133. doi: 10.1038/s41467-019-13795-x (PMC6952388; doi:10.1038/s41467-019-13795-x)
Supplement: Supplementary file 1 — Supplementary Information [file 41467_2019_13795_MOESM1_ESM.pdf]

Supplementary Information

Epidermal control of axonal attachment via ß-spectrin and the GTPase-activating protein TBC-10 prevents axonal degeneration

Coakley et al.

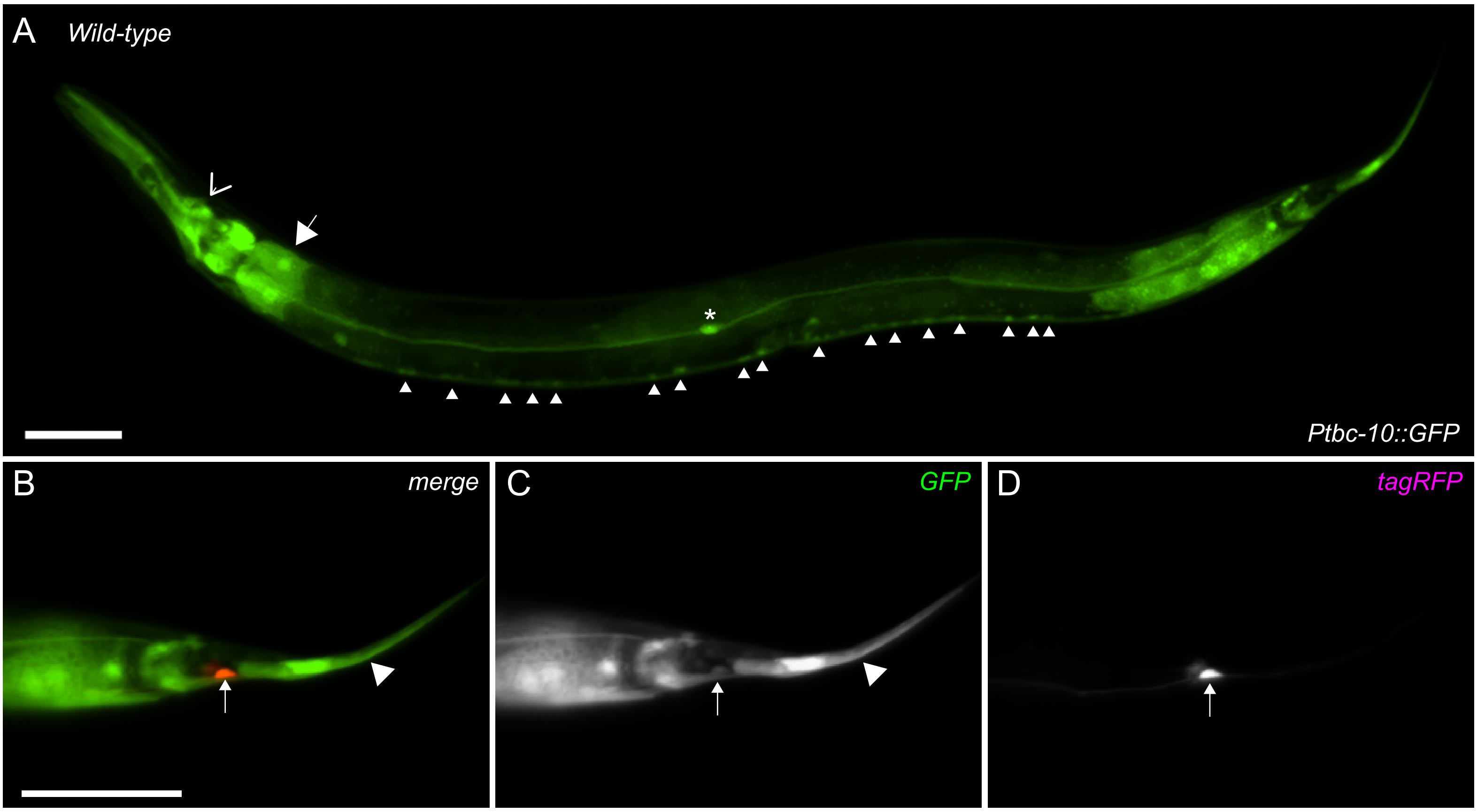

Supplementary Figure 1. ***tbc-10*** **is expressed in multiple tissues, including the PLM neuron.** (A) Image showing the expression pattern of a cytosolic GFP expressed under the control of a 5 kb region of the endogenous *tbc-10* promoter. GFP is visible in multiple tissues, including the excretory canal (asterisk), intestine (arrow), ventral neurons (closed arrowheads) and the pharynx (open arrowhead). (B-D) Magnified image showing weak GFP expression within the PLM neuron (arrow in B-D), which is labeled with a cytosolic tagRFP (*Pmec-17::tagRFP*), as well as in the epidermis (arrowhead in B and C). Expression in these tissues was observed in animals from 10 independent transgenic lines. Scale bars in A and B are 50 μm.

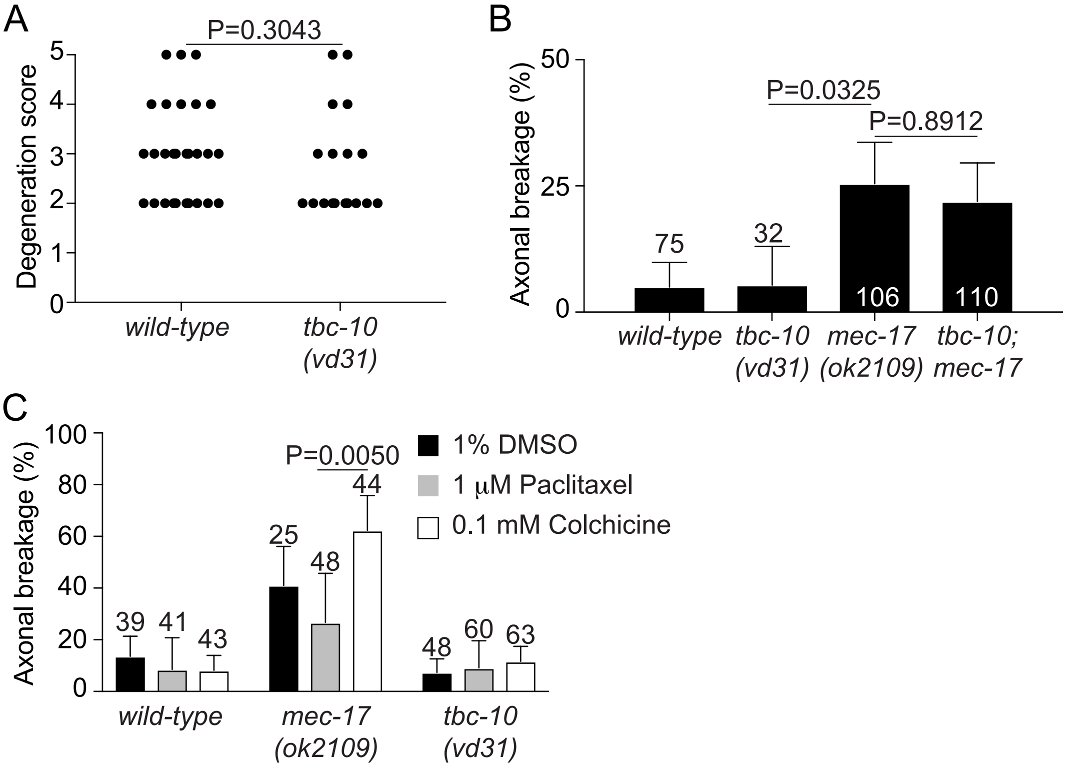

Supplementary Figure 2. TBC-10 does not protect against axonal degeneration after injury. (A) Degeneration of the distal fragment of the PLM axon in wild-type and *tbc-10* mutants following axotomy with a UV-laser. The results are classified as an axonal integrity score with a value of 1 (completely cleared) to 5 (intact). Each data point represents an individual animal. N = 28 animals for wild-type and N = 18 animals for *tbc-10(vd31)* (B) Mean penetrance of axonal breaks in 3-day-old animals carrying single and double mutations in *tbc-10* and the acetyl-transferase *mec-17*. (C) Single mutants for *tbc-10* and *mec-17* were grown on agar plates containing either 1% DMSO, 1 μM paclitaxel, or 0.1 mM colchicine, and scored for axonal breaks at 1-day-old. Mean penetrance of axonal breaks is shown in bar graphs. P-value in A determined by a two-tailed Mann-Whitney test. P-values in B-C determined from a one-way ANOVA with a Tukey multiple-comparison of proportions using an Agresti-Coull interval. Error bars indicate a 95% confidence interval. N-values are indicated on bar graphs and represent the number of individual animals scored for each condition. Source data are provided as a Source Data file.

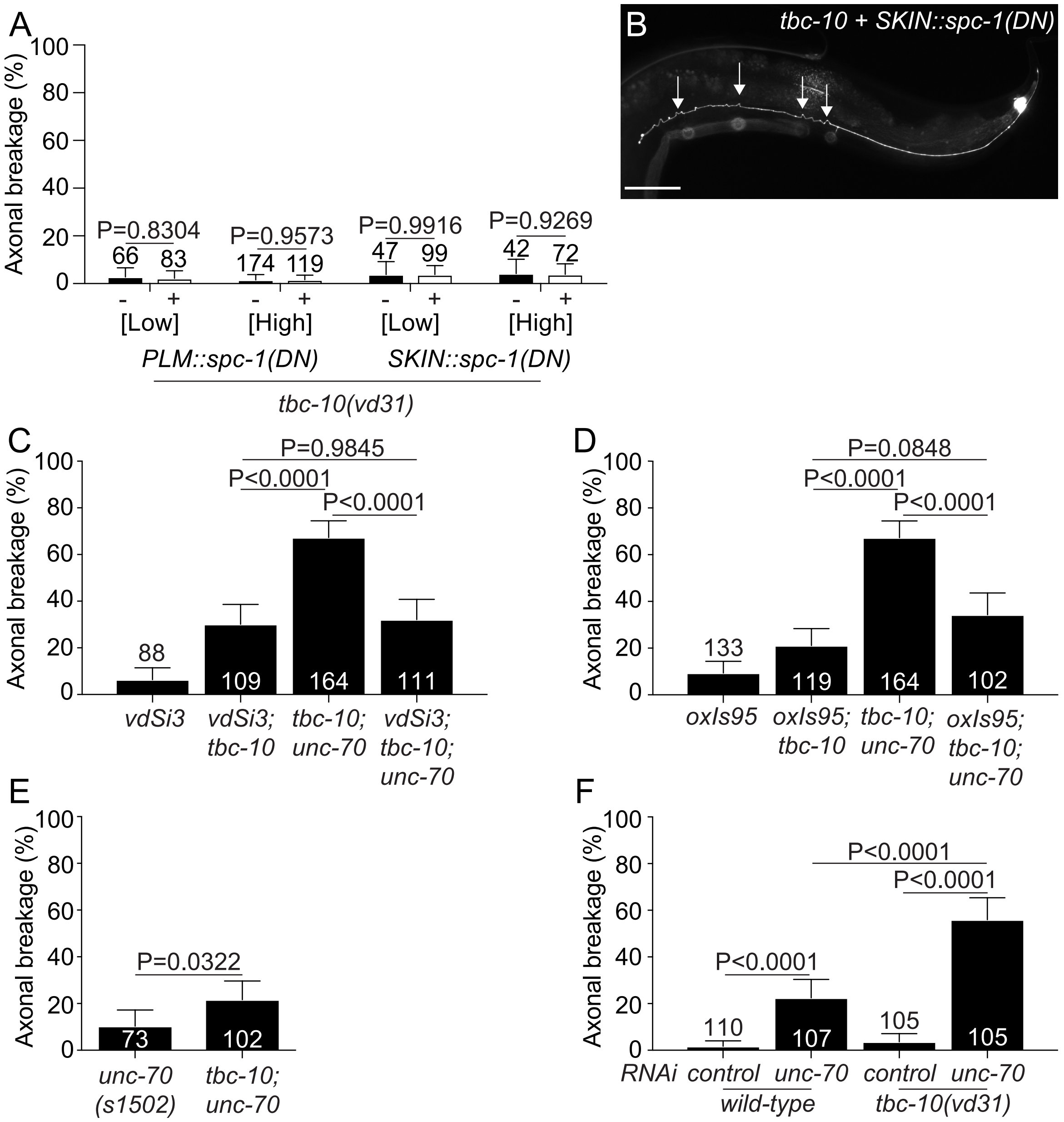

Supplementary Figure 3. UNC-70/ß-spectrin functions in the epidermis to protect from axonal breaks. (A) Mean penetrance of axonal breaks in *tbc-10* mutants carrying transgenes expressing at low or high concentrations of a dominant negative *spc-1/α-spectrin* under either a touch receptor neuron-specific promoter, or an epidermal-specific promoter, versus their respective non-transgenic siblings. (B) Image showing a typical *tbc-10* animal carrying a transgene expressing a dominant negative *spc-1/α-spectrin* under an epidermal-specific promoter, with buckles clearly visible in the PLM axon (indicated by arrows). This phenotype was seen in 8 independent transgenic lines. Scale bar in (B) is 50 μm. (C) Mean penetrance of axonal breaks in *tbc-10* and *unc-70* single and double mutant animals with and without the single-copy insertion *vdSi3(Pdpy-7::unc-70::mKate2)*, which expresses a wild-type copy of UNC-70::mKate2 specifically within the epidermis. (D) Mean penetrance of axonal breaks in *tbc-10* and *unc-70* single and double mutant animals with and without a multi-copy integrated transgene *oxIs95(Ppdi-2::unc-70)*, which overexpresses a wild-type copy of UNC-70 specifically within the epidermis. (E) Mean penetrance of axonal breaks in *unc-70* null mutants and *tbc-10;unc-70* double mutant animals. (F) Mean penetrance of axonal breaks in wild-type and *tbc-10* mutant animals upon *unc-70* RNAi feeding versus their respective controls. P-values in A and E determined from a comparison of proportions using an Agresti-Coull interval. P-values in C, D and F are determined from a one-way ANOVA with a Tukey multiple-comparison of proportions using an Agresti-Coull interval. Error bars indicate a 95% confidence interval. N-values are indicated on bar graphs and represent the number of individual animals scored for each condition. Source data are provided as a Source Data file.

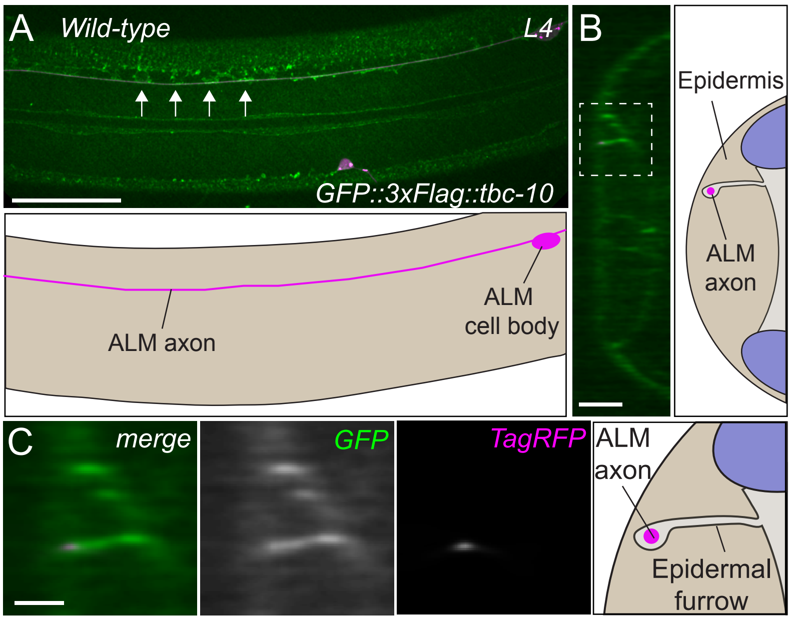

Supplementary Figure 4. TBC-10 localizes to the epidermal membrane. (A) Deconvolved spinning disk confocal maximum projection and scheme of GFP::3xFlag::TBC-10 localization in a typical wild-type animal, with the ALM neuron labeled with a cytosolic TagRFP (*Pmec-17::-tagRFP*; magenta). Arrows indicate regions of the epidermal furrow visible in a lateral perspective. (B) An orthogonal perspective and scheme of GFP::3xFlag::TBC-10 localization in the projected z-stack shown in A. (C) A high magnification image and scheme of the boxed area in B. These micrographs are representative of the GFP::3xFlag::TBC-10 localization seen in N = 6 animals at the L4 stage. Scale bars are 25 µm in A, 5 µm in B, and 2 µm in C.

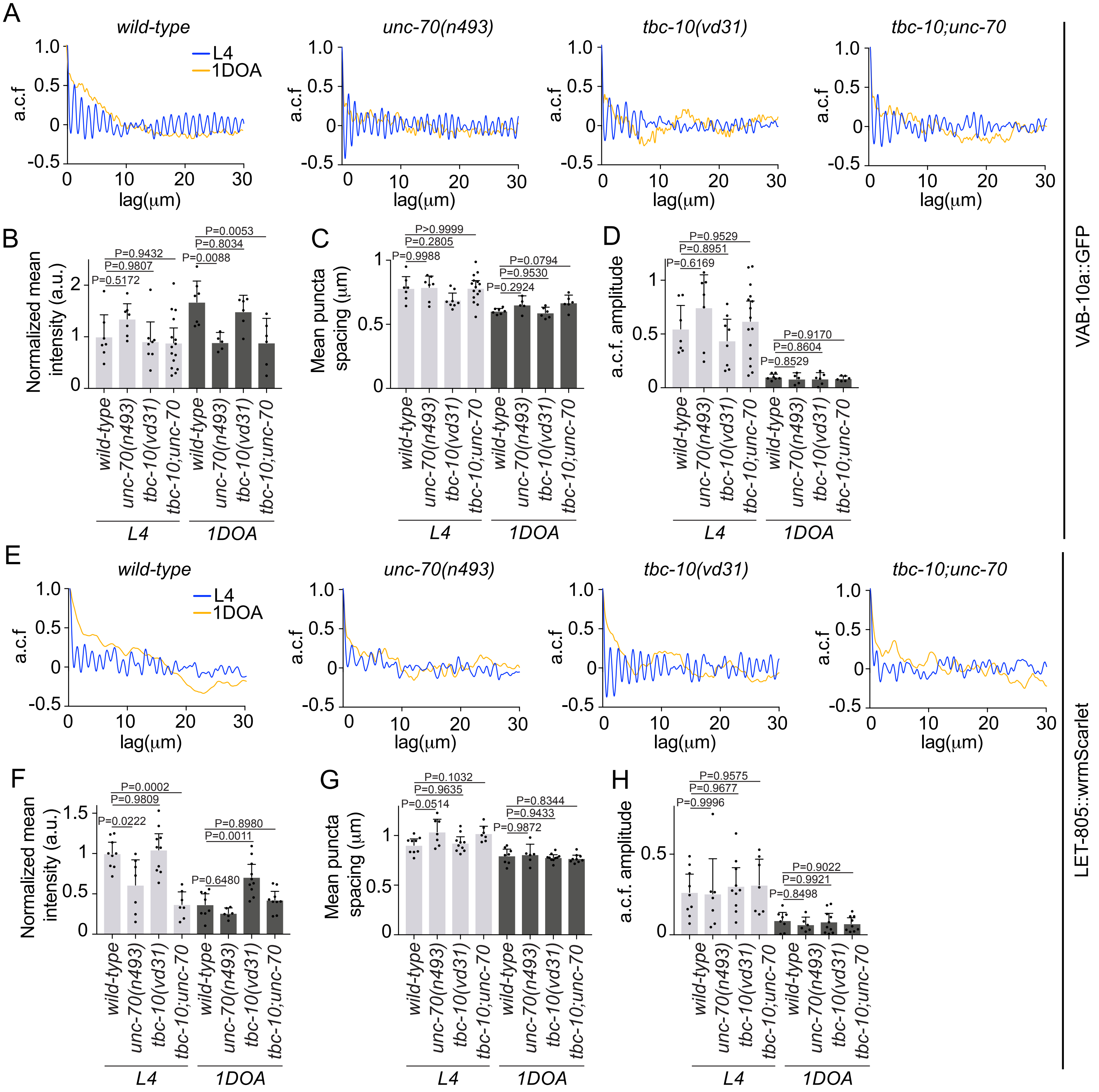

Supplementary Figure 5. Quantification of VAB-10a and LET-805 localization. (A) Autocorrelation analysis of VAB-10a::GFP localizations in representative examples of wild-type, *unc-70*, *tbc-10*, and *tbc-10;unc-70* mutant animals at L4 and 1-day-old adults (1DOAs). (B) Mean intensity of VAB-10a::GFP puncta in wild-type, *unc-70*, *tbc-10*, and *tbc-10;unc-70* mutant animals at L4 and 1DOAs. (C) Mean spacing between VAB-10a::GFP puncta in wild-type, *unc-70*, *tbc-10*, and *tbc-10;unc-70* mutant animals at L4 and 1DOAs. (D) Mean autocorrelation amplitude of VAB-10a::GFP localizations in wild-type, *tbc-10*, *unc-70*, *tbc-10*, and *tbc-10;unc-70* mutant animals at L4 and 1DOAs. (E) Autocorrelation analysis of LET-805::wrmScarlet localizations in representative examples of wild-type, *unc-70*, *tbc-10*, and *tbc-10;unc-70* mutant animals at L4 and 1DOAs. (F) Mean intensity of LET-805::wrmScarlet puncta in wild-type, *unc-70*, *tbc-10*, and *tbc-10;unc-70* mutant animals at L4 and 1DOAs. (G) Mean spacing between LET-805::wrmScarlet puncta in wild-type, *unc-70*, *tbc-10*, and *tbc-10;unc-70* mutant animals at L4 and 1DOAs. (H) Mean autocorrelation amplitude of LET-805::wrmScarlet localizations in wild-type, *unc-70*, *tbc-10*, and *tbc-10;unc-70* mutant animals at L4 and 1DOAs. P-values in B-D and F-H are determined from one-way ANOVAs with a Tukey multiple-comparison of means. Error bars indicate a 95% confidence interval. Each data point on bar graphs represents an individual animal. For panels B-D N = 7, 7, 8 and 15 animals for wild-type, *unc-70*, *tbc-10*, and *tbc-10;unc-70* at the L4 stage and N = 7, 5, 6 and 6 for wild-type, *unc-70*, *tbc-10*, and *tbc-10;unc-70* at the 1DOA stage. For panels F-H N = 9, 7, 10 and 7 animals for wild-type, *unc-70*, *tbc-10*, and *tbc-10;unc-70* at the L4 stage and N = 8, 6, 10 and 9 for wild-type, *unc-70*, *tbc-10*, and *tbc-10;unc-70* at the 1DOA stage. Source data are provided as a Source Data file.

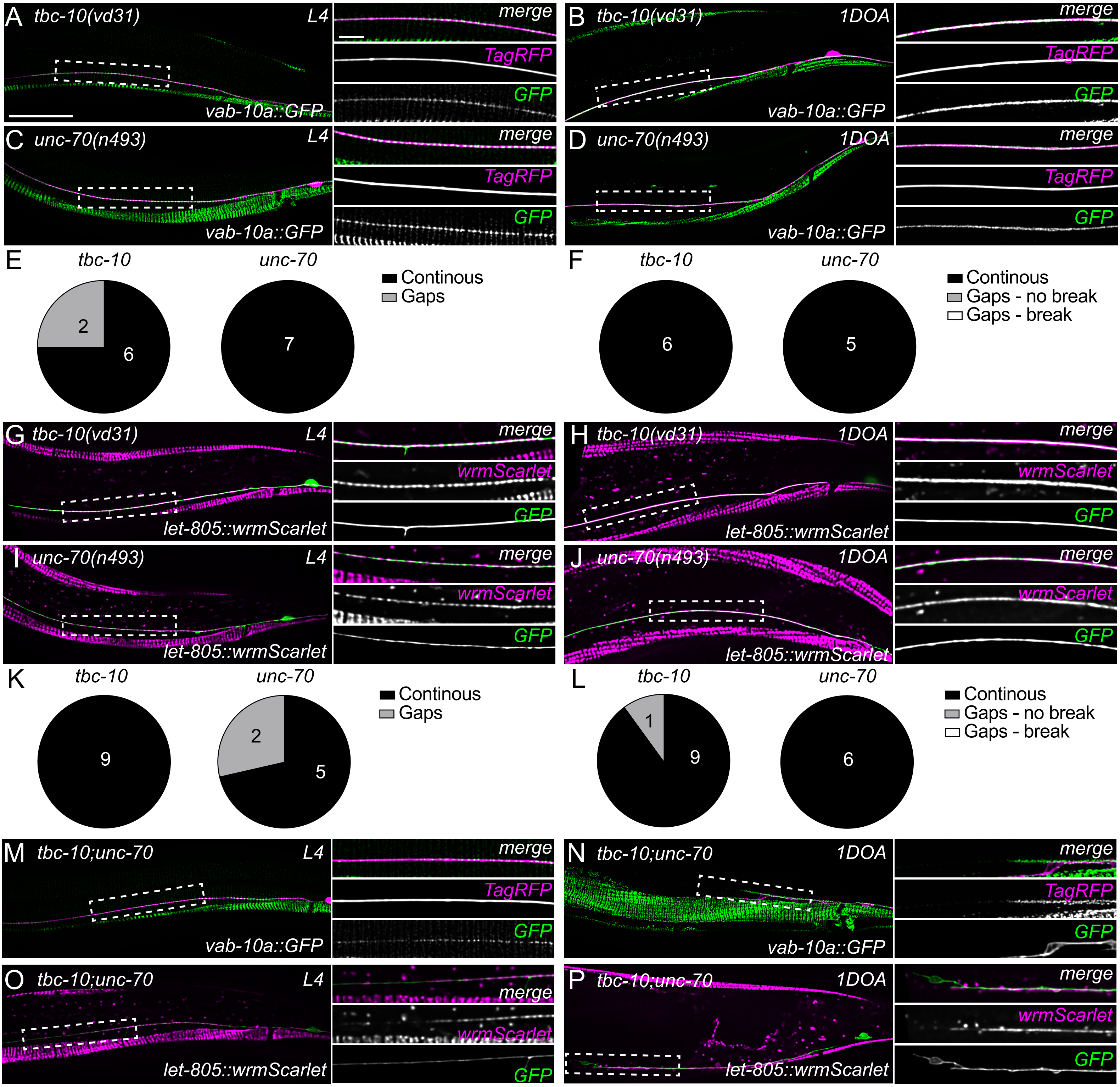

Supplementary Figure 6. Axonal attachment is maintained in single mutants and loss of attachment precedes axonal breakage in *tbc-10;unc-70* mutants. (A-D) Typical endogenous VAB-10a::GFP localization at the L4 (A and C) and 1-day old adult (1DOA) (B and D) stage in *tbc-10* (A-B) and *unc-70* (C-D) mutants from a lateral perspective with the PLM neuron labeled with a cytosolic TagRFP (*Pmec-17::tagRFP*; magenta) similar to as described in Figure 4. (E) Normalized intensities of VAB-10a::GFP along a 50 µm of the PLM axon at the L4 stage were scored as having gaps or continuous attachment. (F) VAB-10a::GFP localization at the 1DOA stage was scored as continuous or containing gaps with, and without, axonal breakage. (G-J) Typical endogenous LET-805::wrmScarlet localization (magenta) at the L4 (G and I) and 1DOA (H and J) stage in *tbc-10* (G-H) and *unc-70* (I-J) mutants from a lateral perspective with the PLM neuron labeled with a GFP (*Pmec-4::GFP*) similar to as described in Figure 5. (K) Normalized intensities of LET-805::wrmScarlet along a 50 µm of the PLM axon at the L4 stage were scored as having gaps or continuous attachment. (L) LET-805::wrmScarlet localization at the 1DOA stage was scored as continuous attachment or containing gaps with, and without, axonal breakage. (M-N) Images of typical endogenous VAB-10a::GFP localization in the same *tbc-10;unc-70* animal (M) at the L4 stage and (N) 24 h later as a 1DOA. The PLM neuron is labeled with a cytosolic tagRFP (*Pmec-17::tagRFP*; magenta). In total 5/10 animals followed from L4 to 1DOA developed breaks, all of these had altered VAB-10a::GFP localization preceding breakage. (O-P) Images of typical endogenous LET-805::wrmScarlet localization in the same *tbc-10;unc-70* animal (O) at the L4 stage and (P) 24 h later as a 1-day-old adult. The PLM neuron is labeled with a cytosolic GFP (*Pmec-4::GFP*). In total 2/4 animals followed from L4 to 1DOA developed breaks, all of these had altered LET-805::wrmScarlet localization preceding breakage. Scale bars in A are 25 µm and 5 µm in the left and side panels, respectively. N-values are indicated on charts and represent the number of individual animals scored for each condition.

Strain	Genotype	Stage	PLM Break	n
CZ10175	*zdIs5*	1DOA	0	227
QH5834	*tbc-10(vd31)*	1DOA	0	163
QH5343	*unc-70(n493);zdIs5*	1DOA	18	309
QH5436	*tbc-10(vd31);unc-70(n493);zdIs5*	1DOA	111	164
QH5878	*tbc-10(tm2907);unc-70(n493);zdIs5*	1DOA	81	138
QH5967	*tbc-10(tm2790);unc-70(n493);zdIs5*	1DOA	26	70
QH5981	*rab-35(b1013); unc-70(n493); zdIs5*	1DOA	3	104
QH5956	*rab-35(b1013); tbc-10(vd31); unc-70(n493); zdIs5*	1DOA	19	130
QH6400	*rme-4(b1001); tbc-10(vd31); unc-70(n493); zdIs5*	1DOA	96	243
QH7764	*flcn-1(ok975); tbc-10(vd31); unc-70(n493); zdIs5*	1DOA	93	155
QH7778	*rme-4(b1001); flcn-1(ok975); tbc-10(vd31); unc-70(n493); zdIs5*	1DOA	39	120
QH6753	*vdSi2(Pdpy-7::unc-70(n493)::mKate2); zdIs5*	1DOA	10	71
QH6693	*vdSi2(Pdpy-7::unc-70(n493)::mKate2); tbc-10(vd31); zdIs5*	1DOA	65	111
QH6858	*vdSi10(Pdpy-7::unc-70(n493)::mKate2); zdIs5*	1DOA	2	45
QH6785	*vdSi10(Pdpy-7::unc-70(n493)::mKate2); tbc-10(vd31); zdIs5*	1DOA	63	74
QH6734	*vdSi3(Pdpy-7::unc-70(WT)::mKate2); zdIs5*	1DOA	4	88
QH6787	*vdSi3(Pdpy-7::unc-70(WT)::mKate2); tbc-10(vd31); zdIs5*	1DOA	32	109
QH7203	*vdSi3(Pdpy-7::unc-70(WT)::mKate2); tbc-10(vd31); unc-70(n493); zdIs5*	1DOA	35	111
QH7034	*vdSi16(Pmec-4::unc-70(n493)::mKate2); tbc-10; zdIs5*	1DOA	3	73
QH7035	*vdSi17(Pmec-4::unc-70(n493)::mKate2); tbc-10; zdIs5*	1DOA	2	74
QH7282	*unc-54(e1009); tbc-10 (vd31); vdSi2[Pdpy-7::unc-70(n493)::mKate2]; zdIs4*	1DOA	5	101
QH7823	*egl-4(ad450); vdSi2(Pdpy-7::unc-70(n493)::mKate2); tbc-10(vd31); zdIs5*	1DOA	52	122
QH7842	*acy-1(ce2); vdSi2[Pdpy-7::unc-70(n493)::mKate2); tbc-10(vd31); zdIs5*	1DOA	69	89
QH6884	*oxIs95(Ppdi-2::unc-70); zdIs5*	1DOA	11	133
QH6885	*oxIs95(Ppdi-2::unc-70); tbc-10(vd31); zdIs5*	1DOA	24	119
QH6886	*oxIs95(Ppdi-2::unc-70); unc-70(n493); zdIs5*	1DOA	2	42
QH5877	*oxIs95(Ppdi-2::unc-70); tbc-10(vd31); unc-70(n493); zdIs5*	1DOA	35	102
CZ10175	*zdIs5*	3DOA	2	75
QH5834	*tbc-10(vd31)*	3DOA	0	32
QH6000	*tbc-10(vd31); mec-17(ok2109); zdIs5*	3DOA	23	110
QH6001	*mec-17(ok2109); zdIs5*	3DOA	26	106
QH6473	*unc-70(s1502); basIs1(Punc-17::unc-70); zdIs5*	1DOA	6	73
QH6476	*tbc-10(vd31); unc-70(s1502); basIs1(Punc-17::unc-70); zdIs5*	1DOA	21	102

Supplementary Table 1.
All genetic mutants and stable transgenic strains used listed together with observed axonal breakage incidence for the PLM neuron.

			Transgenic		Non- Transgenic	
**Strain**	**Genotype**	**Stage**	**PLM Break**	**n**	**PLM Break**	**n**
QH5718	*tbc-10(vd31); unc-70(n493); zdIs5(Pmec-4::GFP); vdEx1211 [Ptbc-10::tbc-10 1 ng/μL]*	1DOA	6	87	65	91
QH5719	*tbc-10(vd31); unc-70(n493); zdIs5(Pmec-4::GFP); vdEx1212 [Ptbc-10::tbc-10 1 ng/μL]*	1DOA	4	134	124	176
QH5731	*tbc-10(vd31); unc-70(n493); zdIs5(Pmec-4::GFP); vdEx1223 [Ptbc-10::tbc-10 1 ng/μL]*	1DOA	4	133	94	142
QH5678	*tbc-10(vd31); unc-70(n493); zdIs5(Pmec-4::GFP); vdEx1188 [Ptbc-10::tbc-10 10 ng/μL]*	1DOA	1	104	31	46
QH5679	*tbc-10(vd31); unc-70(n493); zdIs5(Pmec-4::GFP); vdEx1189 [Ptbc-10::tbc-10 10 ng/μL]*	1DOA	3	108	46	90
QH5680	*tbc-10(vd31); unc-70(n493); zdIs5(Pmec-4::GFP); vdEx1190 [Ptbc-10::tbc-10 10 ng/μL]*	1DOA	2	107	51	103
QH5681	*tbc-10(vd31); unc-70(n493); zdIs5(Pmec-4::GFP); vdEx1191 [Ptbc-10::tbc-10 10 ng/μL]*	1DOA	4	94	56	100
QH5722	*tbc-10(vd31); unc-70(n493); zdIs5(Pmec-4::GFP); vdEx1215 [Pdpy-7::tbc-10 10 ng/μL]*	1DOA	2	101	49	66
QH5724	*tbc-10(vd31); unc-70(n493); zdIs5(Pmec-4::GFP); vdEx1216 [Pdpy-7::tbc-10 10 ng/μL]*	1DOA	0	39	17	48
QH5726	*tbc-10(vd31); unc-70(n493); zdIs5(Pmec-4::GFP); vdEx1218 [Pdpy-7::tbc-10 10 ng/μL]*	1DOA	6	77	41	71
QH5738	*tbc-10(vd31); unc-70(n493); zdIs5(Pmec-4::GFP); vdEx1228 [Pmec-4::tbc-10 5 ng/μL]*	1DOA	60	101	57	95
QH5739	*tbc-10(vd31); unc-70(n493); zdIs5(Pmec-4::GFP); vdEx1229 [Pmec-4::tbc-10 5 ng/μL]*	1DOA	57	99	35	63
QH5717	*tbc-10(vd31); unc-70(n493); zdIs5(Pmec-4::GFP); vdEx1210 [Pmec-4::tbc-10 10 ng/μL]*	1DOA	33	48	42	72
QH5728	*tbc-10(vd31); unc-70(n493); zdIs5(Pmec-4::GFP); vdEx1220 [Pmec-4::tbc-10 10 ng/μL]*	1DOA	40	70	56	101
QH5729	*tbc-10(vd31); unc-70(n493); zdIs5(Pmec-4::GFP); vdEx1221 [Pmec-4::tbc-10 10 ng/μL]*	1DOA	45	101	54	100
QH6017	*tbc-10(vd31); unc-70(n493); zdIs5(Pmec-4::GFP); vdEx1357 [Pmyo-3::tbc-10 10 ng/μL]*	1DOA	22	36	34	49
QH6018	*tbc-10(vd31); unc-70(n493); zdIs5(Pmec-4::GFP); vdEx1358 [Pmyo-3::tbc-10 10 ng/μL]*	1DOA	23	42	53	64
QH6019	*tbc-10(vd31); unc-70(n493); zdIs5(Pmec-4::GFP); vdEx1359 [Pmyo-3::tbc-10 10 ng/μL]*	1DOA	31	48	35	47
QH7053	*tbc-10(vd31); unc-70(n493); zdIs5(Pmec-4::GFP); vdEx1826 [Pdpy-7::htbc1d10a 5 ng/μL]*	1DOA	9	93	68	102
QH7054	*tbc-10(vd31); unc-70(n493); zdIs5(Pmec-4::GFP); vdEx1827 [Pdpy-7::htbc1d10a 5 ng/μL]*	1DOA	10	138	59	94
QH7055	*tbc-10(vd31); unc-70(n493); zdIs5(Pmec-4::GFP); vdEx1828 [Pdpy-7::htbc1d10a 5 ng/μL]*	1DOA	19	85	54	89
QH7367	*tbc-10(vd31); unc-70(n493); zdIs5(Pmec-4::GFP); vdEx2007 [Pdpy-7::htbc1d10b 5 ng/μL]*	1DOA	20	99	64	113
QH7368	*tbc-10(vd31); unc-70(n493); zdIs5(Pmec-4::GFP); vdEx2008 [Pdpy-7::htbc1d10b 5 ng/μL]*	1DOA	24	98	60	115
QH7369	*tbc-10(vd31); unc-70(n493); zdIs5(Pmec-4::GFP); vdEx2009 [Pdpy-7::htbc1d10b 5 ng/μL]*	1DOA	46	124	61	98
QH7370	*tbc-10(vd31); unc-70(n493); zdIs5(Pmec-4::GFP); vdEx2010 [Pdpy-7::htbc1d10b 5 ng/μL]*	1DOA	59	92	51	92
QH7056	*tbc-10(vd31); unc-70(n493); zdIs5(Pmec-4::GFP); vdEx1829 [Pdpy-7::htbc1d10c 5 ng/μL]*	1DOA	68	96	56	82
QH7057	*tbc-10(vd31); unc-70(n493); zdIs5(Pmec-4::GFP); vdEx1830 [Pdpy-7::htbc1d10c 5 ng/μL]*	1DOA	36	76	44	68
QH7058	*tbc-10(vd31); unc-70(n493); zdIs5(Pmec-4::GFP); vdEx1831 [Pdpy-7::htbc1d10c 5 ng/μL]*	1DOA	59	107	31	53
QH7083	*tbc-10(vd31); unc-70(n493); zdIs5(Pmec-4::GFP); vdEx1844 [Pdpy-7::htbc1d10c 5 ng/μL]*	1DOA	52	97	62	94
QH7084	*tbc-10(vd31); unc-70(n493); zdIs5(Pmec-4::GFP); vdEx1845 [Pdpy-7::htbc1d10c 5 ng/μL]*	1DOA	41	118	54	109
QH7085	*tbc-10(vd31); unc-70(n493); zdIs5(Pmec-4::GFP); vdEx1846 [Pdpy-7::htbc1d10c 5 ng/μL]*	1DOA	24	39	16	27
QH7086	*tbc-10(vd31); unc-70(n493); zdIs5(Pmec-4::GFP); vdEx1847 [Pdpy-7::htbc1d10c 5 ng/μL]*	1DOA	40	63	32	58
QH6245	*rab-35(b1013); tbc-10(vd31); unc-70(n493); zdIs5(Pmec-4::GFP); vdEx1454 [Pdpy-7::rab-35cDNA(WT) 0.5 ng/μL]*	1DOA	2	22	5	30
QH6262	*rab-35(b1013); tbc-10(vd31); unc-70(n493); zdIs5(Pmec-4::GFP); vdEx1458 [Pdpy-7::rab-35cDNA(WT) 0.2 ng/μL]*	1DOA	15	104	21	106
QH6263	*rab-35(b1013); tbc-10(vd31); unc-70(n493); zdIs5(Pmec-4::GFP); vdEx1459 [Pdpy-7::rab-35cDNA(WT) 0.2 ng/μL]*	1DOA	98	120	24	87
QH6264	*rab-35(b1013); tbc-10(vd31); unc-70(n493); zdIs5(Pmec-4::GFP); vdEx1460 [Pdpy-7::rab-35cDNA(WT) 0.2 ng/μL]*	1DOA	28	117	16	80
QH6265	*rab-35(b1013); tbc-10(vd31); unc-70(n493); zdIs5(Pmec-4::GFP); vdEx1461 [Pdpy-7::rab-35cDNA(WT) 0.2 ng/μL]*	1DOA	22	86	19	102
QH6224	*rab-35(b1013); tbc-10(vd31); unc-70(n493); zdIs5(Pmec-4::GFP); vdEx1445 [Pdpy-7::rab-35cDNA(S24N) 0.5 ng/μL]*	1DOA	20	41	49	73
QH6225	*rab-35(b1013); tbc-10(vd31); unc-70(n493); zdIs5(Pmec-4::GFP); vdEx1446 [Pdpy-7::rab-35cDNA(S24N) 0.5 ng/μL]*	1DOA	18	34	28	56
QH6227	*rab-35(b1013); tbc-10(vd31); unc-70(n493); zdIs5(Pmec-4::GFP); vdEx1448 [Pdpy-7::rab-35cDNA(S24N) 0.5 ng/μL]*	1DOA	15	57	40	75
QH6228	*rab-35(b1013); tbc-10(vd31); unc-70(n493); zdIs5(Pmec-4::GFP); vdEx1449 [Pdpy-7::rab-35cDNA(S24N) 0.5 ng/μL]*	1DOA	30	41	24	43

QH6266	*rab-35(b1013); tbc-10(vd31); unc-70(n493); zdIs5(Pmec-4::GFP); vdEx1462 [Pdpy-7::rab-35cDNA(S24N) 5 ng/µL]*	1DOA	18	41	30	53
QH6267	*rab-35(b1013); tbc-10(vd31); unc-70(n493); zdIs5(Pmec-4::GFP); vdEx1463 [Pdpy-7::rab-35cDNA(S24N) 5 ng/µL]*	1DOA	27	93	58	87
QH6268	*rab-35(b1013); tbc-10(vd31); unc-70(n493); zdIs5(Pmec-4::GFP); vdEx1464 [Pdpy-7::rab-35cDNA(S24N) 5 ng/µL]*	1DOA	20	63	66	96
QH6269	*rab-35(b1013); tbc-10(vd31); unc-70(n493); zdIs5(Pmec-4::GFP); vdEx1465 [Pdpy-7::rab-35cDNA(S24N) 5 ng/µL]*	1DOA	17	47	53	72
QH7812	*rab-35(b1013); tbc-10(vd31); unc-70(n493); zdIs5(Pmec-4::GFP); vdEx2195[Pdpy-7::rab-35(Q69L)cDNA 0.5 ng/µL]*	1DOA	8	23	12	41
QH7813	*rab-35(b1013); tbc-10(vd31); unc-70(n493); zdIs5(Pmec-4::GFP); vdEx2196[Pdpy-7::rab-35(Q69L)cDNA 0.5 ng/µL]*	1DOA	38	47	21	100
QH7814	*rab-35(b1013); tbc-10(vd31); unc-70(n493); zdIs5(Pmec-4::GFP); vdEx2197[Pdpy-7::rab-35(Q69L)cDNA 0.5 ng/µL]*	1DOA	56	95	40	153
QH7059	*rab-35(b1013); tbc-10(vd31); unc-70(n493); zdIs5(Pmec-4::GFP); vdEx1832 [Pdpy-7::hrab35cDNA(WT) 0.2 ng/µL]*	1DOA	13	72	12	103
QH7060	*rab-35(b1013); tbc-10(vd31); unc-70(n493); zdIs5(Pmec-4::GFP); vdEx1833 [Pdpy-7::hrab35cDNA(WT) 0.2 ng/µL]*	1DOA	15	31	4	41
QH7061	*rab-35(b1013); tbc-10(vd31); unc-70(n493); zdIs5(Pmec-4::GFP); vdEx1834 [Pdpy-7::hrab35cDNA(WT) 0.2 ng/µL]*	1DOA	48	64	12	73
QH7062	*rab-35(b1013); tbc-10(vd31); unc-70(n493); zdIs5(Pmec-4::GFP); vdEx1835 [Pdpy-7::hrab35cDNA(WT) 0.2 ng/µL]*	1DOA	8	70	15	94
QH7787	*tbc-10(vd31); unc-70(n493); zdIs5(Pmec-4::GFP); vdEx2176[Pdpy-7::human_rab-35(S22N)cDNA 0.2 ng/µL]*	1DOA	49	106	106	137
QH7788	*tbc-10(vd31); unc-70(n493); zdIs5(Pmec-4::GFP); vdEx2177[Pdpy-7::human_rab-35(S22N)cDNA 0.2 ng/µL]*	1DOA	47	94	49	68
QH7789	*tbc-10(vd31); unc-70(n493); zdIs5(Pmec-4::GFP); vdEx2178[Pdpy-7::human_rab-35(S22N)cDNA 0.2 ng/µL]*	1DOA	75	117	32	48
QH7790	*tbc-10(vd31); unc-70(n493); zdIs5(Pmec-4::GFP); vdEx2179[Pdpy-7::human_rab-35(S22N)cDNA 0.2 ng/µL]*	1DOA	39	64	41	69
QH7791	*tbc-10(vd31); unc-70(n493); zdIs5(Pmec-4::GFP); vdEx2180[Pdpy-7::human_rab-35(S22N)cDNA 0.2 ng/µL]*	1DOA	39	60	30	52
QH7815	*rab-35(b1013); (tbc-10(vd31); unc-70(n493); zdIs5(Pmec-4::GFP); vdEx2198[Pdpy-7::human_rab-35(Q67L)cDNA 0.2 ng/µL]*	1DOA	89	145	29	162
QH7816	*rab-35(b1013); (tbc-10(vd31); unc-70(n493); zdIs5(Pmec-4::GFP); vdEx2199[Pdpy-7::human_rab-35(Q67L)cDNA 0.2 ng/µL]*	1DOA	43	119	10	78
QH7817	*rab-35(b1013); (tbc-10(vd31); unc-70(n493); zdIs5(Pmec-4::GFP); vdEx2200[Pdpy-7::human_rab-35(Q67L)cDNA 0.2 ng/µL]*	1DOA	7	34	7	45
QH7818	*rab-35(b1013); (tbc-10(vd31); unc-70(n493); zdIs5(Pmec-4::GFP); vdEx2201[Pdpy-7::human_rab-35(Q67L)cDNA 0.2 ng/µL]*	1DOA	6	55	10	58
QH7819	*rab-35(b1013); (tbc-10(vd31); unc-70(n493); zdIs5(Pmec-4::GFP); vdEx2202[Pdpy-7::human_rab-35(Q67L)cDNA 0.2 ng/µL]*	1DOA	3	43	11	77
QH6524	*tbc-10(vd31); zdIs5(Pmec-4::GFP); vdEx1537[Pdpy-7::spc-1(1-170)::mCherry 5 ng/µL]*	1DOA	0	44	0	9
QH6525	*tbc-10(vd31); zdIs5(Pmec-4::GFP); vdEx1538[Pdpy-7::spc-1(1-170)::mCherry 5 ng/µL]*	1DOA	2	49	0	35
QH6526	*tbc-10(vd31); zdIs5(Pmec-4::GFP); vdEx1539[Pdpy-7::spc-1(1-170)::mCherry 5 ng/µL]*	1DOA	1	66	0	30
QH6528	*tbc-10(vd31); zdIs5(Pmec-4::GFP); vdEx1541[Pdpy-7::spc-1(1-170)::mCherry 5 ng/µL]*	1DOA	2	42	0	50
QH6529	*tbc-10(vd31); zdIs5(Pmec-4::GFP); vdEx1542[Pdpy-7::spc-1(1-170)::mCherry 5 ng/µL]*	1DOA	2	99	0	47
QH6530	*tbc-10(vd31); zdIs5(Pmec-4::GFP); vdEx1543[Pdpy-7::spc-1(1-170)::mCherry 10 ng/µL]*	1DOA	2	69	0	28
QH6531	*tbc-10(vd31); zdIs5(Pmec-4::GFP); vdEx1544[Pdpy-7::spc-1(1-170)::mCherry 10 ng/µL]*	1DOA	0	63	0	38
QH6534	*tbc-10(vd31); zdIs5(Pmec-4::GFP); vdEx1547[Pdpy-7::spc-1(1-170)::mCherry 10 ng/µL]*	1DOA	1	72	0	42
QH6519	*tbc-10(vd31); zdIs5(Pmec-4::GFP); vdEx1532[Pmec-17::spc-1(1-170)::mCherry 5 ng/µL]*	1DOA	0	83	0	66
QH6520	*tbc-10(vd31); zdIs5(Pmec-4::GFP); vdEx1533[Pmec-17::spc-1(1-170)::mCherry 5 ng/µL]*	1DOA	0	58	1	76
QH6521	*tbc-10(vd31); zdIs5(Pmec-4::GFP); vdEx1534[Pmec-17::spc-1(1-170)::mCherry 10 ng/µL]*	1DOA	1	174	0	119
QH6522	*tbc-10(vd31); zdIs5(Pmec-4::GFP); vdEx1535[Pmec-17::spc-1(1-170)::mCherry 10 ng/µL]*	1DOA	0	68	0	40
QH6523	*tbc-10(vd31); zdIs5(Pmec-4::GFP); vdEx1536[Pmec-17::spc-1(1-170)::mCherry 10 ng/µL]*	1DOA	0	216	1	104
QH6657	*tbc-10(vd31); zdIs5(Pmec-4::GFP); vdEx1603[Pdpy-7::spc-1(1-170)::mCherry 5ng/µL + Pmec-17::spc-1(1-170)::mCherry 5 ng/µL]*	1DOA	2	73	1	14
QH6658	*tbc-10(vd31); zdIs5(Pmec-4::GFP); vdEx1604[Pdpy-7::spc-1(1-170)::mCherry 5ng/µL + Pmec-17::spc-1(1-170)::mCherry 5 ng/µL]*	1DOA	0	33	0	40
QH6659	*tbc-10(vd31); zdIs5(Pmec-4::GFP); vdEx1605[Pdpy-7::spc-1(1-170)::mCherry 5ng/µL + Pmec-17::spc-1(1-170)::mCherry 5 ng/µL]*	1DOA	2	66	0	25
QH6660	*tbc-10(vd31); zdIs5(Pmec-4::GFP); vdEx1606[Pdpy-7::spc-1(1-170)::mCherry 5ng/µL + Pmec-17::spc-1(1-170)::mCherry 5 ng/µL]*	1DOA	2	48	1	19
QH6661	*tbc-10(vd31); zdIs5(Pmec-4::GFP); vdEx1607[Pdpy-7::spc-1(1-170)::mCherry 10ng/µL + Pmec-17::spc-1(1-170)::mCherry 10 ng/µL]*	1DOA	2	65	0	46
QH6662	*tbc-10(vd31); zdIs5(Pmec-4::GFP); vdEx1608[Pdpy-7::spc-1(1-170)::mCherry 10ng/µL + Pmec-17::spc-1(1-170)::mCherry 10 ng/µL]*	1DOA	0	44	0	11
QH6663	*tbc-10(vd31); zdIs5(Pmec-4::GFP); vdEx1609[Pdpy-7::spc-1(1-170)::mCherry 10ng/µL + Pmec-17::spc-1(1-170)::mCherry 10 ng/µL]*	1DOA	0	41	0	66
QH6664	*tbc-10(vd31); zdIs5(Pmec-4::GFP); vdEx1610[Pdpy-7::spc-1(1-170)::mCherry 10ng/µL + Pmec-17::spc-1(1-170)::mCherry 10 ng/µL]*	1DOA	1	21	0	23
QH6665	*tbc-10(vd31); zdIs5(Pmec-4::GFP); vdEx1611[Pdpy-7::spc-1(1-170)::mCherry 10ng/µL + Pmec-17::spc-1(1-170)::mCherry 10 ng/µL]*	1DOA	1	73	0	40

Supplementary Table 2.
All semi-stable transgenic strains used listed together with observed axonal breakage incidence for the PLM neuron in transgenic and non-transgenic siblings.

Plasmid	Feature	Backbone	Insert	Reagents	Cloning method	Enzymes	Source
pSC8	Ptbc-10::gfp	pNV Punc-25::GFP	PCR generated<br>gDNA template	Ptbc-10 F 5'-gttcgagcggccgcaatttgatattagcgtcatc-3'<br>Ptbc-10 R 5'-ctgcaggctagcctggaagaaaaacgggcata-3'	Restriction digest	NotI/NheI	This work
pSC11	Pdpy-7::tbc-10	Pdpy-7::unc-54(3'UTR)	PCR generated<br>gDNA template	tbc-10 F 5'-atcagcgctagcatgcaaatctcatccacctc-3'<br>tbc-10 R 5'-gacgctggtacctcagtcaaatctcatttgtc-3'	Restriction digest	NheI/KpnI	This work
pSC12	Ptbc-10::tbc-10	pSC11 Pdpy-7::tbc-10	pSC8 Ptbc-10::gfp	-	Restriction digest	HindIII/NheI	This work
pSC13	Pmec-4::tbc-10	pSC11 Pdpy-7::tbc-10	Pmec-4::unc-54(3'UTR)	-	Restriction digest	BamHI/HindIII	This work
pSC14	Pmyo-3::tbc-10	pSC11 Pdpy-7::tbc-10	Pmyo-3::unc-54(3'UTR)	-	Restriction digest	BamHI/HindIII	This work
pFR8	Pdpy-7::tbc1d10a	Pdpy-7::unc-54(3'UTR)	gBlock (IDT)	tbc1d10a (NM_031937) with 30bp homology regions<br>5'-taacatagaacattttcaggaggacccttggctagc-3' at the 5' end<br>5'- ggtaccggtgaccaactgagcgccggtcgctaccat-3' at the 3' end	Gibson assembly	NheI/KpnI	This work
pKG1	Pdpy-7::tbc1d10b	Pdpy-7::unc-54(3'UTR)	PCR generated<br>(nested)<br>OHu07035 template	tbc1d10b outer F 5'-tgggaggtctatataagcagag-3'<br>tbc1d10b outer R 5'-tagaaggcacagtcgagg-3'<br>tbc1d10b inner F 5'-attttcaggaggacccttggatggagacgggcacggcg-3'<br>tbc1d10b inner R 5'- gctcagttggtcaccggtactcagaagtaagcgtcctgccg-3'	Gibson assembly	NheI/KpnI	This work
pFR9	Pdpy-7::tbc1d10c	Pdpy-7::unc-54(3'UTR)	gBlock (IDT)	tbc1d10c (NM_198517; codon optimized) with 30bp homology regions<br>5'-taacatagaacattttcaggaggacccttggctagc-3' at the 5' end<br>5'- ggtaccggtgaccaactgagcgccggtcgctaccat-3' at the 3' end	Gibson assembly	NheI/KpnI	This work
OHu07035	tbc1d10b	-	-	-	-	-	Genscript (USA)
mg356	Pmec-17::spc-1(DN)::mCherry	-	-	-	-	-	Miriam Goodman
pSC22	Pdpy-7::spc-1(DN)::mCherry	Pdpy-7::unc-54(3'UTR)	mg356 Pmec-17::spc-1(DN)::mCherry	-	Restriction digest	BamHI/PacI	This work
pSC19	Pdpy-7::rab-35	pSC11 Pdpy-7::tbc-10	PCR generated<br>pID3.01::rab-35 template	rab-35 F 5'-gacatcgctagcatggcgggaactcgggattacg-3'<br>rab-35 R 5'-gacgctggtaccttatccacatttgcacttcttttc-3'	Restriction digest	NheI/KpnI	This work
pSC20	Pdpy-7::rab-35(S24N)	pSC11 Pdpy-7::tbc-10	PCR generated<br>pID3.01::rab-35(S24N) template	rab-35 F 5'-gacatcgctagcatggcgggaactcgggattacg-3'<br>rab-35 R 5'-gacgctggtaccttatccacatttgcacttcttttc-3'	Restriction digest	NheI/KpnI	This work
pSC21	Pdpy-7::rab-35(Q69L)	pSC11 Pdpy-7::tbc-10	PCR generated<br>pID3.01::rab-35(Q69L) template	rab-35 F 5'-gacatcgctagcatggcgggaactcgggattacg-3'<br>rab-35 R 5'-gacgctggtaccttatccacatttgcacttcttttc-3'	Restriction digest	NheI/KpnI	This work
pID3.01::rab-35	rab-35	-	-	-	-	-	Barth Grant
pID3.01::rab-35(S24N)	rab-35(S24N)	-	-	-	-	-	Barth Grant
pID3.01::rab-35(Q69L)	rab-35(Q69L)	-	-	-	-	-	Barth Grant
pFR7	Pdpy-7::hrab35	Pdpy-7::unc-54(3'UTR)	gBlock (IDT)	rab35 (NM_006861) with 30bp overhangs<br>5'-taacatagaacattttcaggaggacccttggctagc-3' at the 5' end<br>5'- ggtaccggtgaccaactgagcgccggtcgctaccat-3' at the 3' end	Gibson assembly	NheI/KpnI	This work
pFR10	Pdpy-7::hrab35(Q67L)	Pdpy-7::hrab35	-	hrab35 Q67L QCF 5'-ggaagcgctccagccccgctgtg-3'<br>hrab35 Q67L QCR 5'-cacagcggggctggagcgcttcc-3'	Site-directed Mutagenesis	-	This work
pFR11	Pdpy-7::hrab35(S22N)	Pdpy-7::hrab35	-	hrab35 S22N QCF 5'-cgcaacagtaaactgttcttgcccacaccgc-3'<br>hrab35 S22N QCR 5'-gcggtgtgggcaagaacagtttactgttgcg-3'	Site-directed Mutagenesis	-	This work
pSC23	Pdpy-7::mKate::rab-35(WT)	PCR generated<br>pSC19 Pdpy-7::rab-35(WT) template	PCR generated<br>pSJ9061Pmyo-3:mKate2 template	vector F 5'-catgttctccttgatgagctcggagaccatggatccttatctggaacaaaatgtaagaat-3'<br>vector R 5'-gcccgttactgcgacctcctacaagctcgagcgggaactcgggattacgatcatttgttc-3'<br>insert F 5'-attcttacatttgttccagataaggatccatggtctccgagctcatcaaggagaacatg-3'<br>insert R 5'-gaacaaatgatcgtaatcccgagttcccgctcgagcttgtaggaggtcgcagtaacgggc-3'			This work
pSJ9061	Pmyo-3:mKate2	-	-	-	-	-	Steven Zuryn
pSC15	Ptbc10::gfp-c1^sec^3xflag::tbc-10	pDD282 GFP^SEC^3xFlag	PCR generated<br>gDNA template	5' arm F 5'-acgttgtaaaacgacggccagtcgccggcacaactccgcgcagattaga-3'<br>5' arm R 5'-tccagtgaacaattcttctcctttactcatctggaagaaaaacgggcataact-3'<br>3' arm F 5'-cgtgattacaaggatgacgatgacaagagaatgcaaatctcatcaacctca-3'<br>3' arm R 5'-tcacacaggaaacagctatgaccatgttatacatttgggccttccacgat-3'	Gibson assembly	ClaI/SpeI	This work
pDD282	GFP^SEC^3xFlag	-	-	-	-	-	Bob Goldstein
pSC16	Peft-3::cas9 + tbc-10 sgRNA	pDD162 Peft-3::cas9	-	tbc-10 sgRNA F 5'-agatgtcttggtagttgccatttgatgagggttttagagctagaa-3'<br>tbc-10 sgRNA R 5'-gctctaaaaccctcatcaaatggcaactaccaagacatctcgcaa-3'	Site-directed Mutagenesis	-	This work
pDD162	Peft-3::cas9	-	-	-	-	-	Bob Goldstein
pGH8	Prab-3::mCherry	-	-	-	-	-	Erik Jorgensen
pCFJ104	Pmyo-3::mCherry	-	-	-	-	-	Erik Jorgensen
pCFJ90	Pmyo-2::mCherry	-	-	-	-	-	Erik Jorgensen
pSC24	Pdpy-7::unc-70	Pdpy-7::unc-54(3'UTR)	PCR generated<br>PK133 venus::unc-70 template	unc-70 F 5'-agctaccccgggatggctacggtcgacggcaattcaggcgcc-3'<br>unc-70 R 5'-ctagcagctagccttcttgcctcgagagaaaaatc-3'	Restriction digest	XmaI/NheI	This work
PK133	venus::unc-70	-	-	-	-	-	Kang Shen
pSC25	Pdpy-7::unc-70(DN)	pSC24 Pdpy-7::unc-70	-	unc-70(DN) F 5'-cgaagaaaccatcaaacccatcaagaaacacgagg-3'<br>unc-70(DN) R 5'-cctcgtgtttcttgatgggtttgatggtttcttcg-3'	Site-directed Mutagenesis	-	This work
pSC26	Pdpy-7::unc-70::mKate2	pSC24 Pdpy-7::unc-70	PCR generated<br>pSJ9061Pmyo-3:mKate2 template	mKate2 F 5'-ctagcagctagcgtctccgagctcatcaaggag-3'<br>mKate2 R 5'-gtcaccggtaccttataggaggtcgcagtaacgggcg-3'	Restriction digest	NheI/KpnI	This work
pSC27	Pdpy-7::unc-70(DN)::mKate2	pSC25 Pdpy-7::unc-70(DN)	PCR generated<br>pSJ9061Pmyo-3:mKate2 template	mKate2 F 5'-ctagcagctagcgtctccgagctcatcaaggag-3'<br>mKate2 R 5'-gtcaccggtaccttataggaggtcgcagtaacgggcg-3'	Restriction digest	NheI/KpnI	This work
pSC30	Minimal-Mos1-NeoR-Pdpy-7::unc-70::mKate2	PCR generated<br>pCFJ910 Minimal-Mos1-NeoR template	PCR generated<br>pSC26 Pdpy-7::unc-70::mKate2 template	miniMos F 5'-tactatgaaactcatctcacaacgaagcttgtatctagaaccggtgacgtcaccatggga-3'<br>miniMos R 5'-acattcccaatataccaaacataactgtttctaggtgagctctggtaccctctagtcaag-3'<br>Pdpy-7 insert F 5'-tcccatggtgacgtcaccggttctagatacaagcttcgttgtgagatgagtttcatagta-3'<br>Pdpy-7 insert R 5'-cttgactagagggtaccagagctcacctagaaacagttatgtttggtatattgggaatgt-3'	Gibson assembly	-	This work
pSC31	Minimal-Mos1-NeoR-Pdpy-7::unc-70(DN)::mKat	PCR generated<br>pCFJ910 Minimal-Mos1-NeoR template	PCR generated<br>pSC27 Pdpy-7::unc-70(DN)::mKate2 templa	miniMos F 5'-tactatgaaactcatctcacaacgaagcttgtatctagaaccggtgacgtcaccatggga-3'<br>miniMos R 5'-acattcccaatataccaaacataactgtttctaggtgagctctggtaccctctagtcaag-3'<br>Pdpy-7 insert F 5'-tcccatggtgacgtcaccggttctagatacaagcttcgttgtgagatgagtttcatagta-3'<br>Pdpy-7 insert R 5'-cttgactagagggtaccagagctcacctagaaacagttatgtttggtatattgggaatgt-3'	Gibson assembly	-	This work
pCFJ910	Minimal-Mos1-NeoR	-	-	-	-	-	Erik Jorgensen
pSC28	Pmec-4::unc-70::mKate2	Pmec-4::unc-54(3'UTR)	pSC26 Pdpy-7::unc-70::mKate2	-	Restriction digest	KpnI/XmaI	This work
pSC32	Minimal-Mos1-NeoR-Pmec-4::unc-70::mKate2	PCR generated<br>pCFJ910 Minimal-Mos1-NeoR template	PCR generated<br>pSC28 Pmec-4::unc-70::mKate2 template	miniMos F 5'-tactatgaaactcatctcacaacgaagcttgtatctagaaccggtgacgtcaccatggg-3'<br>miniMos R 5'- acattcccaatataccaaacataactgtttctaggtgagctctggtaccctctagtcaag-3'<br>Pmec-4 insert F 5'-tcccatggtgacgtcaccggttctagatacagcttcaatacaagctcaaatacaatacta-3'<br>Pmec-4 insert R 5'-cttgactagagggtaccagagctcacctagaaacagttatgtttggtatattgggaatg-3'	Gibson assembly	-	This work
pSC37	Mos1-NeoR-Pmec-4::unc-70(DN)::mKate2	pSC32 Minimal-Mos1-NeoR-Pmec-4::unc-70::mKat	-	unc-70(DN) F 5'-cgaagaaaccatcaaacccatcaagaaacacgagg-3'<br>unc-70(DN) R 5'-cctcgtgtttcttgatgggtttgatggtttcttcg-3'	Site-directed Mutagenesis	-	This work
pMA122	Phsp16.41:peel-1	-	-	-	-	-	Erik Jorgensen
pCFJ601	Peft-3:mos1	-	-	-	-	-	Erik Jorgensen

Supplementary Table 3.

Details of the construction and source of all plasmids generated and used in this work.
