# Supplementary material for: Epidermal control of axonal attachment via β-spectrin and the GTPase-activating protein TBC-10 prevents axonal degeneration
Source: Nat Commun. 2020 Jan 9;11:133. doi: 10.1038/s41467-019-13795-x (PMC6952388; doi:10.1038/s41467-019-13795-x)
Supplement: Supplementary file 2 — Reporting Summary [file 41467_2019_13795_MOESM2_ESM.pdf]

nature research

Corresponding author(s): Massimo A. Hilliard

Last updated by author(s): Oct 30, 2019

Reporting Summary

Nature Research wishes to improve the reproducibility of the work that we publish. This form provides structure for consistency and transparency in reporting. For further information on Nature Research policies, see Authors & Referees and the Editorial Policy Checklist .

Statistics

For all statistical analyses, confirm that the following items are present in the figure legend, table legend, main text, or Methods section.

n/a	Confirmed	
☐	☒	The exact sample size (*n*) for each experimental group/condition, given as a discrete number and unit of measurement
☒	☐	A statement on whether measurements were taken from distinct samples or whether the same sample was measured repeatedly
☐	☒	The statistical test(s) used AND whether they are one- or two-sided<br>*Only common tests should be described solely by name; describe more complex techniques in the Methods section.*
☒	☐	A description of all covariates tested
☐	☒	A description of any assumptions or corrections, such as tests of normality and adjustment for multiple comparisons
☐	☒	A full description of the statistical parameters including central tendency (e.g. means) or other basic estimates (e.g. regression coefficient) AND variation (e.g. standard deviation) or associated estimates of uncertainty (e.g. confidence intervals)
☐	☒	For null hypothesis testing, the test statistic (e.g. *F*, *t*, *r*) with confidence intervals, effect sizes, degrees of freedom and *P* value noted<br>*Give P values as exact values whenever suitable.*
☒	☐	For Bayesian analysis, information on the choice of priors and Markov chain Monte Carlo settings
☒	☐	For hierarchical and complex designs, identification of the appropriate level for tests and full reporting of outcomes
☒	☐	Estimates of effect sizes (e.g. Cohen's *d*, Pearson's *r*), indicating how they were calculated

Our web collection on statistics for biologists contains articles on many of the points above.

Software and code

Policy information about availability of computer code

Data collection	N/A
Data analysis	Custom Matlab (R2015a) code was used to analyse the periodicity, intensity of puncta in Figure S5.

For manuscripts utilizing custom algorithms or software that are central to the research but not yet described in published literature, software must be made available to editors/reviewers. We strongly encourage code deposition in a community repository (e.g. GitHub). See the Nature Research guidelines for submitting code & software for further information.

Data

Policy information about availability of data

All manuscripts must include a data availability statement. This statement should provide the following information, where applicable:

- Accession codes, unique identifiers, or web links for publicly available datasets
- A list of figures that have associated raw data
- A description of any restrictions on data availability

The datasets generated and analysed for Figures 1, 3, 6 and Supplementary Figures 2 and 3 are available in the Source Data file. Datasets generated and analysed for Figures 4, 5, and 6 and Supplementary Figures 5 and 6 are available from the corresponding author on reasonable request.

Field-specific reporting

Please select the one below that is the best fit for your research. If you are not sure, read the appropriate sections before making your selection.

☒ Life sciences ☐ Behavioural & social sciences ☐ Ecological, evolutionary & environmental sciences

October 2018

For a reference copy of the document with all sections, see nature.com/documents/nr-reporting-summary-flat.pdf

Life sciences study design

All studies must disclose on these points even when the disclosure is negative.

Sample size	The sample size used here was based on our previous published results, which indicate a sufficient power to detect differences between wild-type and other mutants. The sample size reflects the nature of the assay and it is also used in most leading studies on neuronal development and maintenance in C. elegans.
Data exclusions	The only animals that were excluded from the analysis were those animals that died following axotomy, because axonal degeneration could not be confirmed in these animals (Figure S2A). This criterion was pre-established as a condition for the scoring of axonal degeneration. In rare cases animals also perished following RNAi knockdown of unc-70 (Figure S3F). These animals were excluded from scoring axonal breaks. This criterion was pre-established as a condition for the scoring of axonal breaks.
Replication	The expression of any transgene from semi-stable extrachromosomal array was replicated in multiple independent lines. Where presented in the main figures a representative strain is depicated and the raw data for all replicates are presented in Supplementary Table 2.
Randomization	No specific randomization was used. The animal groups were determined by their genotype (wild-type, mutant, transgenic, double mutant, triple mutant), with each population considered isogenic. A number of animals from each population was taken from the petri dish with no bias (the first one in the field of view) other than age (L4 stage) and sex (hermaphrodites).
Blinding	C. elegans wild-type and a large set of mutant animals were used in this study. The genotypes of the animals was known to the investigator and was often associated with an obvious visible phenotype making blinding impractical.

Reporting for specific materials, systems and methods

We require information from authors about some types of materials, experimental systems and methods used in many studies. Here, indicate whether each material, system or method listed is relevant to your study. If you are not sure if a list item applies to your research, read the appropriate section before selecting a response.

Materials & experimental systems

n/a	Involved in the study	
x		Antibodies
x		Eukaryotic cell lines
x		Palaeontology
	x	Animals and other organisms
x		Human research participants
x		Clinical data

Methods

n/a	Involved in the study	
x		ChIP-seq
x		Flow cytometry
x		MRI-based neuroimaging

Animals and other organisms

Policy information about studies involving animals; ARRIVE guidelines recommended for reporting animal research

Laboratory animals	Caenorhabditis elegans
Wild animals	The study did not involve wild animals.
Field-collected samples	The study did not include field-collected samples.
Ethics oversight	No ethical approval was required.

Note that full information on the approval of the study protocol must also be provided in the manuscript.
